# A group of nuclear factor Y transcription factors are sub-functionalized during endosperm development in monocots

**DOI:** 10.1093/jxb/ery087

**Published:** 2018-03-05

**Authors:** Zhiguo E, Tingting Li, Huaya Zhang, Zehou Liu, Hui Deng, Sandeep Sharma, Xuefeng Wei, Lei Wang, Baixiao Niu, Chen Chen

**Affiliations:** 1 Key Laboratory of Rice Biology, China National Rice Research Institute, Hangzhou, China; 2 Jiangsu Key Laboratory of Crop Genetics and Physiology, Co-Innovation Center for Modern Production Technology of Grain Crops, Key Laboratory of Plant Functional Genomics of the Ministry of Education, Yangzhou University, Yangzhou, China; 3 Chengdu Institute of Biology, Chinese Academy of Sciences, Chengdu, China; 4 Marine Biotechnology and Ecology Division, CSIR-Central Salt & Marine Chemicals Research Institute, Bhavnagar, Gujarat, India

**Keywords:** Endosperm, nuclear factor Y transcription factor, phylogenic analysis, protein interaction, rice, transcription factors

## Abstract

Nuclear factor Y (NF-Y) is a heterotrimeric transcription factor that consists of three subunits, NF-YA, NF-YB, and NF-YC. Gene functions of NF-Ys during endosperm development are not well understood. In this study, we identified eight rice NF-Y-encoding genes, namely *OsNF-YA8*, *OsNF-YB1*,*9*, and *OsNF-YC8*,*9*,*10*,*11*,*12*, that are predominantly expressed in the endosperm. Interestingly, the close homologs of these OsNF-Ys are present only in monocot species and are also preferentially expressed in the endosperm, suggesting that they have roles in the regulation of endosperm development. A systemic analysis of interactions between rice endosperm-preferential NF-Ys in yeast revealed that OsNF-YBs and OsNF-YCs could interact with each other. We also found that the endosperm-preferential OsNF-YBs and OsNF-YCs could interact with some ethylene response factors (ERFs) of rice. Unlike OsNF-YC8,9,10, the members of OsNF-YB1,9 or OsNF-YC 11,12 showed no transcriptional activation when present alone. However, they displayed functional activity while in dimer form. In addition, *OsNF-YB1*-knockout lines showed significant changes in seed morphology, further confirming its role in endosperm development. Our findings provide evidence that a group of phylogenetically conserved NF-Ys is probably differentiated in monocots to regulate endosperm development.

## Teaser

A group of phylogenetically conserved nuclear factor Y transcription factors are preferentially expressed in the endosperm and probably play essential roles in endosperm development for rice and other monocots.

## Introduction

Rice (*Oryza sativa*) provides most of the calories consumed by human beings globally. The endosperm is the edible part of rice and other cereals. After double-fertilization, the primary rice endosperm nucleus develops into a syncytium, a cell with multiple free nuclei that is formed by cell division but uncoupled from cytokinesis (Wu *et al.*, 2016). Simultaneous cellularization of the free nuclei initiates proliferation of endosperm cells. After several rounds of mitotic division, endosperm cells occupy a central vacuole and start to accumulate starch (Wu *et al.*, 2016). Between two to five layers of the outermost cells of the endosperm are differentiated into aleurone cells while the inner cells are differentiated into storage cells (Zhou *et al.*, 2013). The pattern of endosperm development in other monocots is similar to that of rice (Olsen, 2001; Leroux *et al.*, 2014; Zhang *et al.*, 2016*a*).

Several groups of transcription factors, such as the MADS-box genes *OsMADS6* (Zhang *et al.*, 2010), *OsMADS29* (Yin and Xue, 2012), and *OsMADS87* (Chen *et al.*, 2016), the bZIP family gene *RISBZ1* (Yamamoto *et al.*, 2006; Kawakatsu *et al.*, 2009), the DOF family gene *RPBF* (Yamamoto *et al.*, 2006; Kawakatsu *et al.*, 2009), and the WRKY family gene *OsWRKY78* (Zhang *et al.*, 2011), are essential for rice endosperm and seed development. Nuclear factor Y (NF-Y), also known as Heme Activator Protein or CCAAT-binding factor, is conserved across kingdoms (Petroni *et al.*, 2012; Laloum *et al.*, 2013). NF-Ys consist of three families: NF-YA, NF-YB, and NF-YC. As a result of possessing of histone fold domains (HFDs), NF-YB and NF-YC can form a heterodimer, which generates a surface for the NF-YA accession to form a NF-YA/NF-YB/NF-YC trimeric complex (Nardone *et al.*, 2017; Gnesutta *et al.*, 2017). The CCAAT-motif binding ability of NF-YA (Laloum *et al.*, 2013; Gnesutta *et al.*, 2017) and the transcriptional activation activities of NF-YB and NF-YC (Coustry *et al.*, 1995, 1996) allow the heterotrimeric complex to act as a transcription factor. The NF-Y complex may further interact with other transcription factors to regulate expression of downstream targets (Yamamoto *et al.*, 2009; Cao *et al.*, 2014; Huang *et al.*, 2015; Xu *et al.*, 2016). Interestingly, whilst there are only one or two encoding genes of each NF-Y family in mammals and yeast (Dolfini *et al.*, 2012), plants have substantially expanded their NF-Y genes (Laloum *et al.*, 2013). As an example, the rice genome encodes 11 NF-YAs, 11 NF-YBs, and 12 NF-YCs (Yang *et al.*, 2016). The expansion of plant NF-Ys may increase the number of possible NF-Y complexes and probably contributes to neo-functionalization or sub-functionalization of the complexes. For instance, a group of phylogenetically related NF-Ys that are predominantly expressed in nodules are specifically involved in nodulation in legumes (Baudin *et al.*, 2015).

Several NF-Ys have been found to be indispensable for seed development. *Leafy Cotyledon 1* (*LEC1*), also known as *NF-YB9* of Arabidopsis (*AtNF-YB9*), and its homolog *LEC1-like* (*L1L* or *AtNF-YB6*) are required for embryo maturation (Kwong *et al.*, 2003; Lee *et al.*, 2003). LEC1 and L1L belong to a phylogenetically conserved clade of plants (Xie *et al.*, 2008). Rice NF-YB7 (OsNF-YB7) and OsNF-YB9 are the most similar homologs of LEC1 in rice. *OsNF-YB7*/*OsHAP3E* is expressed in the developing embryo and callus (Thirumurugan *et al.*, 2008). Ectopic expression of *OsNF-YB7* results in defects in vegetative and reproductive development (Ito *et al.*, 2011; Zhang and Xue, 2013). However, due to the lethality caused by RNA interference-mediated gene silencing of *OsNF-YB7* (Ito *et al.*, 2011; Zhang and Xue, 2013), its role in embryogenesis and seed development remains to be elucidated. *OsNF-YB1*, a gene specifically expressed in the endosperm, can co-ordinate with NF-YC members to regulate cell proliferation, grain filling, and sugar-loading of the endosperm (Sun *et al.*, 2014; Bai *et al.*, 2015; Xu *et al.*, 2016). Bai *et al.* (2015) showed that OsNF-YB1 was able to recognize the CCAAT motifs of some sucrose transporters, while Xu *et al.* (2016) suggested that OsNF-YB1 probably lacks CCAAT-box binding activity but that it could bind to ERF transcription factors in the endosperm to regulate gene expression. In addition, OsNF-YB1 is able to interact with OsMADS18, a MADS-box family transcription factor of rice (Masiero *et al.*, 2002). The biological significance of the interaction is yet to be determined.

Some NF-Ys are found to be preferentially expressed in rice, wheat, and maize endosperm (Stephenson *et al.*, 2007; Yang *et al.*, 2016; Zhang *et al.*, 2016*b*); however, the importance of NF-Ys for endosperm development is not well understood. In this study, we performed a comprehensive analysis with endosperm-preferential NF-Ys in rice and identified two phylogenetically conserved NF-YB clades and one NF-YC clade in monocots. The endosperm-specific gene expression and the interactions with other endosperm-preferential NF-Y members suggest that these family genes have specific roles in endosperm development. The study provides novel insights into the functional role of NF-Ys in rice seed development.

## Materials and methods

### Plant material and growth conditions

Rice plants (*Oryza sativa* subsp. *Japonica*, cv Kitaake) were grown in a greenhouse with regular water supply and nutrient management. Various tissues, including the leaf blade, leaf sheath, flag leaf, stem, and young panicles at the booting stage were collected for RNA isolation. Before flowering, the plants were moved into a growth chamber that was maintained at a consistent 28 °C with 12/12 h light/dark cycle and 50% humidity. The seeds were labeled when flowering. Caryopses of different ages were collected and immediately frozen by liquid nitrogen for RNA isolation. The barley variety Morex was planted in an experimental plot in Chengdu, China, in 2016. Various barley tissues were collected at the flowering stage. The seeds were labeled on the day of flowering to enable sampling of caryopses of different ages. At 25 d after fertilization, endosperms and embryos were carefully separated to avoid contamination of maternal tissues.

### Generation of mutant plants

The targets for CRISPR/Cas9-mediated gene targeting were designed using the web-based tool CRISPR Primer Designer (http://plantsignal.cn/CRISPR/crispr_primer_designer.html). The targets were then cloned into the vector BGK032 using the CRISPR/Cas9 vector constructing kit (Biogle). All the constructs were transformed into the *Agrobacterium* strain EHA105 and used for plant transformation as described previously (Chen *et al.*, 2014). The DNA fragments embracing the targets of the plant transformants were amplified for sequencing. The T_0_ and T_1_ homozygous mutants of *osnf-yb1*, *osnf-yb7*, *osnf-yc8*, *osnf-yc11*, and *osnf-yc12* were obtained for phenotypic analysis.

### RNA extraction and real-time PCR assay

Total RNA from the different plant tissues was isolated using a Plant RNA Kit (Omega) following the manufacturer’s protocol. Total RNA was treated with an RNA-free DNase set (Omega). cDNA was synthesized by oligo-dT primers using PrimeScript RT Master Mix (TaKaRa). The standard procedure provided by the manufacturer was used for the reactions. A 2-μl sample of the diluted cDNA was used for real-time PCR in a 20 μl reaction using the AceQ^®^ qPCR SYBR^®^ Green Master Mix (Vazyme, China). The real-time PCR reactions were performed on a CFX Connect™ Real-Time System (BioRad). Three independent biological replicates were set for each sample. A rice proteasome gene (LOC_Os03g63430) was used as an internal control. Quantification of the relative expression was calculated by the ΔΔ*C*_T_ method (Livak and Schmittgen, 2001). Primers used for the real-time PCR reactions can be found in Supplementary Table S1 at *JXB* online.

### Expression analysis of endosperm-preferential NF-Ys in monocots

We used data deposited in Genevestigator® to conduct an expression analysis of rice NF-Ys. A hierarchical clustering analysis of the expression of rice NF-Ys was also performed with the similarity search tool provided by Genevestigator®. Expression of the NF-Ys during the early caryopsis developmental stages was obtained from RiceXPro (http://ricexpro.dna.affrc.go.jp). Expression data for the *OsNF-YB1*,*7*,*9*-*like* and *OsNF-YC8*,*11-like* genes of maize and sorghum were obtained from the Expression Atlas (https://www.ebi.ac.uk/gxa/home). For genes with no data in the Expression Atlas, we searched for their expression through the Maize eFP Browser (http://bar.utoronto.ca/efp_maize/cgi-bin/efpWeb.cgi) or the MOROKOSHI Sorghum transcriptome database (http://sorghum.riken.jp/morokoshi/Home.html).

### Alignment, phylogenetic analysis, and modeling of protein structure

The core domains of OsNF-YA8, OsNF-YB1,7,9, and OsNF-YC8,11 were used as queries for BLAST (https://blast.ncbi.nlm.nih.gov/Blast.cgi) to identify their close homologs in other plant species. The protein and DNA sequences of the endosperm-preferential NF-Ys were obtained from BioMart of the Phytozome database (www.phytozome.jgi.doe.gov/biomart/martview/). Alignment was conducted using Clustal Omega (www.ebi.ac.uk/Tools/msa/clustalo/). The phylogenetic neighbor-joining trees were generated using MEGA7.0 (Kumar *et al.*, 2016). The intensive model of the Phyre2 web portal was used for modeling the NF-Ys (Kelley *et al.*, 2015).

### Yeast two-hybrid assay

The coding sequences of *OsNF-YA8*, *OsNF-YB1*,*7*,*9*, *OsNF-YC8*,*9*,*10*,*11*,*12*, and *OsERF72*,*74*,*114*,*115* were amplified by high-fidelity DNA polymerase GXL (Takara) from endosperm cDNA using the primers listed in Supplementary Table S1. The genes were cloned into either pGBK-T7 or pGAD-T7 using the ClonExpress® II One Step Cloning Kit (Vazyme). The prey and bait plasmids were transformed into the yeast strains Y187 and Y2HGold, respectively, using the Frozen-EZ Yeast Transformation II Kit™ (Zymo). After mating of the two strains, co-transformants were selected on SD–L–T plates. Interactions were tested using SD–L–T–H with an optimized content of 3-AT and SD–L–T–H–A medium, simultaneously.

### Transcriptional activation assay in yeast

The coding sequences of *OsNF-YA8*, *OsNF-YB1*,*7*,*9*, and *OsNF-YC8*,*9*,*10*,*11*,*12* were cloned into pGBK-T7. We amplified the GAL4 activation domain from a pGAD-T7 empty vector and fused it with GAL4 DNA-binding domain using the ClonExpress® II One Step Cloning Kit (Vazyme). The construct was used as a positive control for transcriptional activation activity. We cloned *OsNF-YB1* and *OsNF-YC12* respectively into the multiple cloning site 1 (MCS1) and the MCS2 of the vector pBridge (Clontech). All of the constructs were then transformed into yeast strain Y2HGold to investigate the activity of transcriptional activation on SD–T/–H/–A medium. The primers used in the experiment are listed in Supplementary Table S1.

### Sub-cellular localization analysis

The coding sequences of *OsNF-YA8*, *OsNF-YB1*,*7*,*9*, and *OsNF-YC8*,*9*,*10*,*11*,*12* were cloned into the binary vectors p1300-LV, pHB-GFP, or pHB-mCherry using the ClonExpress® II One Step Cloning Kit (Vazyme). The constructs were transformed into *Agrobacterium tumefaciens* strain GV3101 and infiltrated into tobacco (*Nicotiana benthamiana*) leaf epidermal cells to transiently express the NF-Y::Venus, NF-Y::GFP, or NF-Y::mCherry recombinant fusions. About 48 h after infiltration, the fluorescence signals were examined using a confocal laser scanning microscope (LSM710, Zeiss). Primer information is listed in Supplementary Table S1.

### Bimolecular fluorescence complementation (BiFC) and split luciferase complementation assays

The coding sequences of *OsNF-YA8* and *OsNF-YB9* were cloned into pCAMBIA1300S-YN and pCAMBIA2300S-YC, respectively, to fuse with the N- and C-terminals of yellow fluorescence protein (nYFP and cYFP) and transformed into *Agrobacterium tumefaciens* strain GV3101. In addition, HAL3, which can dimerize in the cytoplasm, was cloned into pCAMBIA1300S-YN and pCAMBIA2300S-YC as a positive control (Su *et al.*, 2016). The constructs, *nYFP-YA8* and *cYFP-YB9*, *nYFP-OsHAL3* and *cYFP-OsHAL3*, and *nYFP-5790* and *cYFP-5790*, were co-infiltrated in tobacco leaves for about 48 h. The YFP fluorescence signals were examined using a confocal laser scanning microscope (LSM710, Zeiss).

Similarly, *OsNF-YA8* and *OsNF-YB9* was cloned into the binary vectors JW771 and JW772, respectively, to fuse with the N- and C-terminals of luciferase (nLUC and cLUC). *nLUC-OsNF-YA8* and *cLUC-OsNF-YB9* together with the combinations of *cLUC* and *nLUC*, *nLUC-OsNF-YA8* and *cLUC*, and *nLUC* and *cLUC-OsNF-YB9*, were transiently expressed in tobacco leaves for about 48 h. The Luciferase Assay System (Promega) was used to detect the interactions. The chemiluminescence signal was detected using the Tanon Imaging System (5200 Multi, Tanon).

## Results

### Endosperm-preferentially expressed NF-Ys in rice

**Fig. 1.**
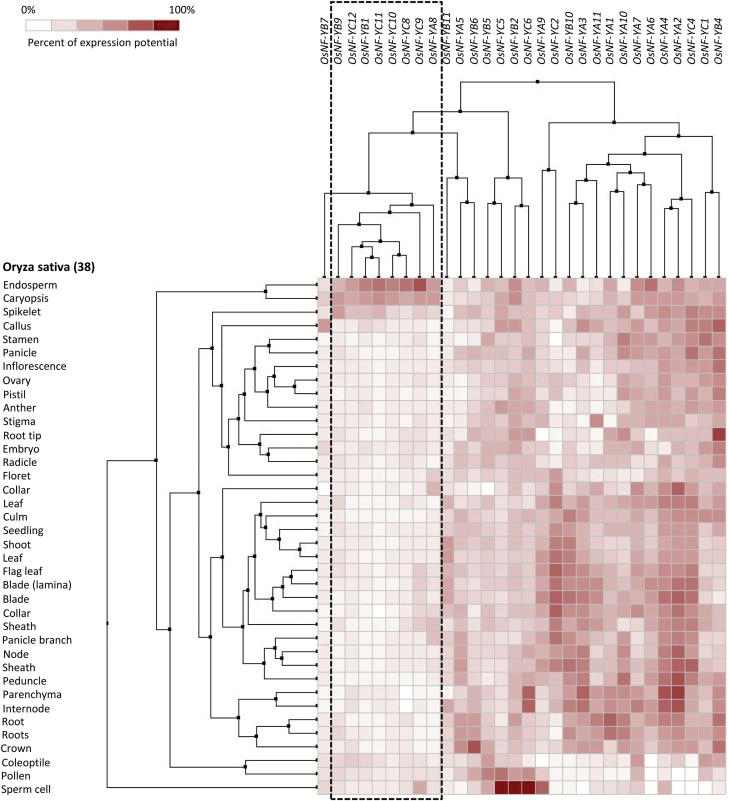
Cluster analysis of the expression of rice NF-Ys in various tissues. A group of rice *NF-Ys* (boxed by dashed lines), namely *OsNF-YA8*, *OsNF-YB1*, *OsNF-YB9*, *OsNF-YC8*, *OsNF-YC9*, *OsNF-YC10*, *OsNF-YC11*, and *OsNF-YC12*, are predominantly expressed in the endosperm.

**Fig. 2.**
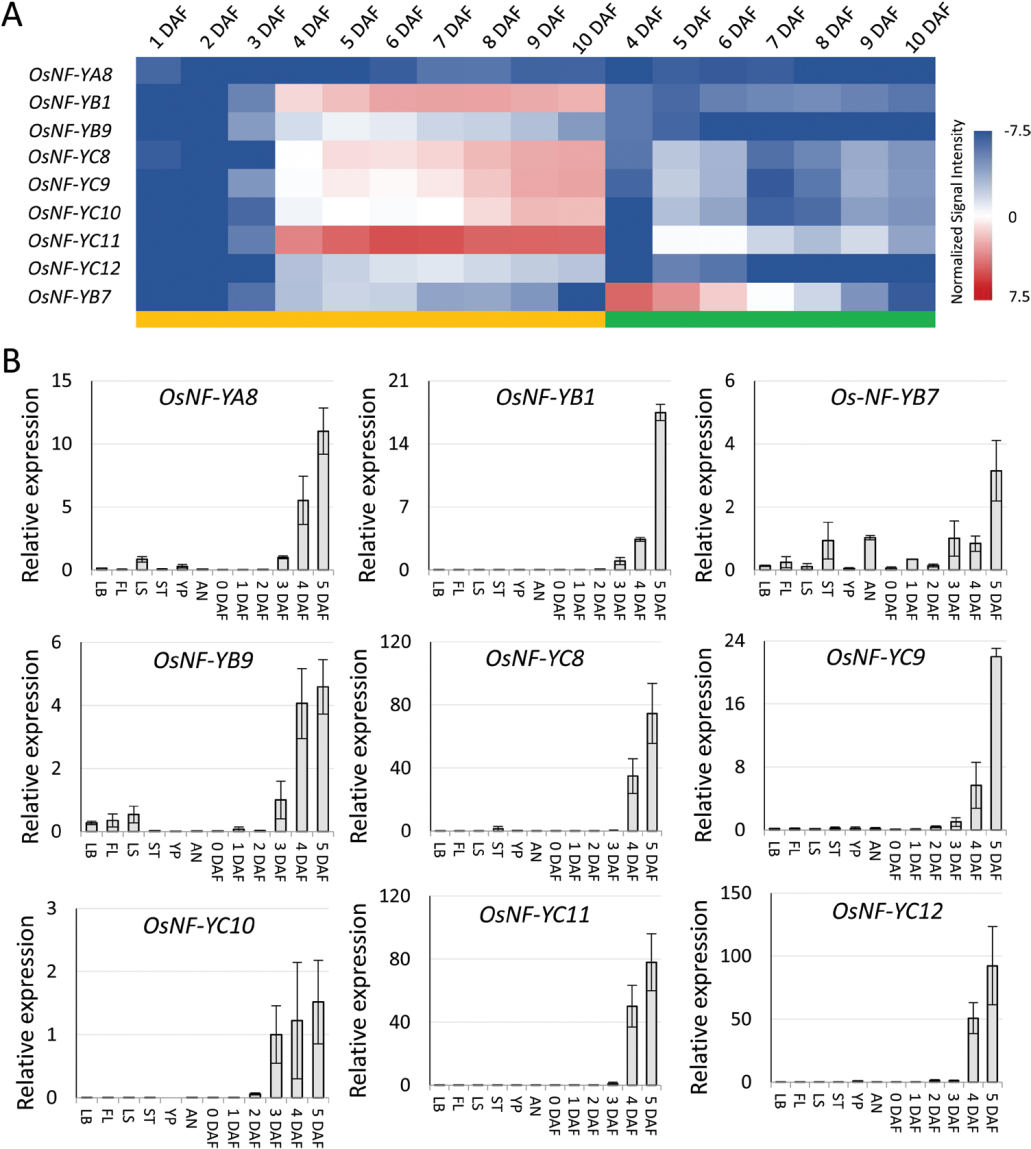
Activation of the seed-preferential *OsNF-Y*s after fertilization. (A) Heat map of the expression of *OsNF-YA8, OsNF-YB1, OsNF-YB7, OsNF-YB9, OsNF-YC8, OsNF-YC9, OsNF-YC10, OsNF-YC11,* and *OsNF-YC12* at different days after fertilization (DAF). The yellow and green bars at the bottom indicate the ovary (including seed coat, endosperm, and embryo) and the embryo, respectively. The data were obtained from the RiceXPro database. (B) Confirmation of the caryopsis-preferential expression patterns and gene activation after fertilization by real-time PCR. Expression is relative to that of the rice proteasome gene LOC_Os03g63430, which was used as an internal control. LB, leaf blade; FL, flag leaf; LS, leaf sheath; ST, stem; YP, young panicle; AN, anther. 0 DAF indicates unfertilized ovary; 1–5 DAF indicate ovaries at different ages. Three biological replicates were used for analysis; data are means (±SD).

The rice genome encodes 11 NF-YAs, 11 NF-YBs, and 12 NF-YCs (Yang *et al.*, 2016). The cluster analysis of their expression profiles showed that a group of NF-Ys, including *OsNF-YA8*, *OsNF-YB1*,*9*, and *OsNF-YC8*,*9*,*10*,*11*,*12*, were predominantly expressed in the rice endosperm (Figs 1, 2A). We further confirmed the expression pattern of these NF-Ys by real-time PCR. All endosperm-preferential NF-Ys were activated between 3 to 4 d after fertilization (DAF) (Fig. 2B). The results showed that each NF-Y family had members predominantly expressed in rice endosperm. Using a laser capture microdissection (LCM) assay, Xu *et al.* (2016) separated aleurone cells from 8-DAF rice caryopses for RNA-seq. From this data, we found that *OsNF-YB1* and *OsNF-YB9* were highly expressed in aleurone cells, while *OsNF-YA8* and *OsNF-YCs* were moderately expressed (Supplementary Fig. S1A). *OsNF-YC11* and some other NF-YC members were abundant in 8-DAF endosperm of Kitaake (Supplementary Fig. S1B), implying that the *OsNF-YC*s are probably highly expressed in the central starchy endosperm.

There is another rice NF-YB gene, *OsNF-YB7*, that also showed a pattern of preferentially expression in the caryopsis (Fig. 1); however, the microarray data (Fig. 2A) and a previous study (Thirumurugan *et al.*, 2008) suggested that its expression was mainly restricted to the embryo rather than the endosperm.

### Phylogenetic analysis of endosperm-preferential NF-Y rice genes

To further study the evolution of endosperm-preferential NF-Ys, we performed sequence alignment and phylogenetic analyses. We used the core sequences of the conserved domains for further analysis because the ones outside are highly variable. In comparison with other NF-YA members of rice, OsNF-YA8 showed several differences in the core sequence, many of which were located within the A2 domain (Supplementary Fig. S2A, D). Using the core sequence of OsNF-YA8 as a query search, we failed to identify its close homologs in other plant species, which indicated that OsNF-YA8 might have evolved after the divergence of the *Oryza* genus.

**Fig. 3.**
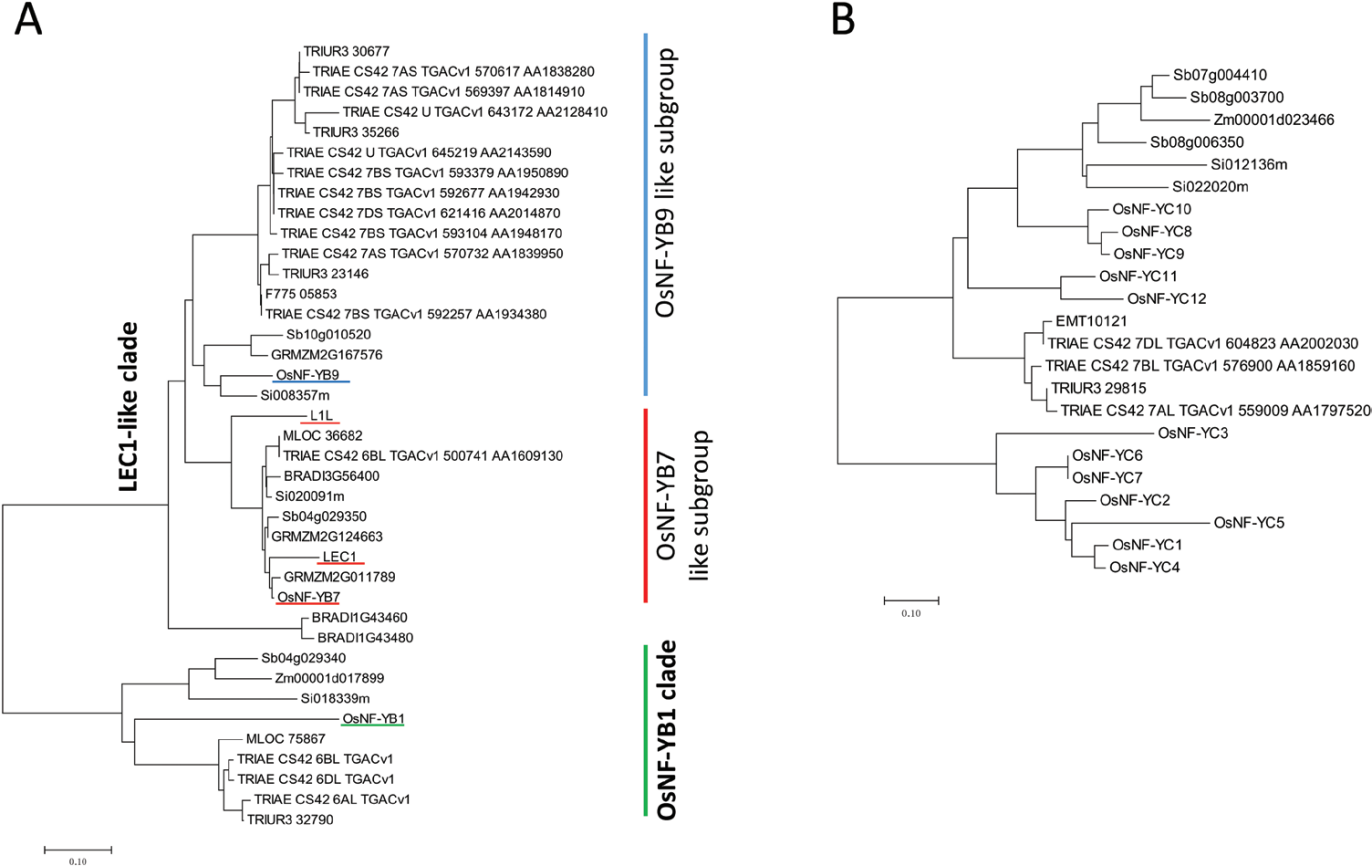
Neighbor-joining phylogenetic trees of the endosperm-preferential OsNF-YBs and OsNF-YCs. (A) Phylogenetic tree of OsNF-YB1, OsNF-YB7, OsNF-YB9, and their homologs. (B) Phylogenetic tree of OsNF-YCs and the close homologs of OsNF-YC8 and OsNF-YC12. TRIUR, *Triticum urartu*; TRIAE, *Triticum aestivum*; F775, *Aegilops tauschii*; Sb, *Sorghum bicolor*; GRMZM(ZM), *Zea mays*; Os, *Oryza sativa*; Si, *Setaria italica*; At, *Arabidopsis thaliana*; MLOC *Hordeum vulgare*; BRADI, *Brachypodium distachyon*. The core sequences of the conserved domains of NF-YBs and NF-YCs were used for construction of the trees. (This figure is available in colour at *JXB* online.)

An alignment analysis of the NF-YB family members showed that the core sequence of OsNF-YB1 was distinct from the others (Supplementary Fig. S2B, E). Many of these variations were present within the α2 domain, which is believed to be involved in NF-YA and NF-YC interactions with NF-YBs (Petroni *et al.*, 2012; Laloum *et al.*, 2013). Close homologs of OsNF-YB1 could be only found in monocots (Supplementary Fig. S3). The core sequences of OsNF-YB9 and OsNF-YB7, two LEC1 homologs of rice, were very similar (Supplementary Fig. S2B). The Asp at position 84 and the His at position 79 of L1L are diagnostic for LEC1 family members and are essential for gene function (Gnesutta *et al.*, 2017). The Asp and His residues were conserved in OsNF-YB9 and OsNF-YB7 (Supplementary Fig. S2B). Interestingly, our phylogenetic analysis showed that the LEC1 homologs in monocots could be divided into two phylogenetic clades (Supplementary Fig. S4A). OsNF-YB7 was included in the same group of LEC1 and L1L, whereas OsNF-YB9 belonged to the other clade that consisted exclusively of monocot homologs (Fig. 3A and Supplementary Fig. S4A). Three substitutions could be found by comparing the core sequences of the OsNF-YB7-like (OsYB7L) and OsNF-YB9-like (OsYB9L) proteins in different monocot species (Supplementary Fig. S4B). Hydrophobic Ala, Val, or Leu in OsYB9Ls substituted the residue Thr at position 33 of OsYB7Ls. The residues QREQ at position 49–52 were highly conserved in OsYB7Ls but were variable in OsYB9Ls. In addition, the Tyr or Phe at position 89 of OsYB7Ls was substituted by Met in OsYB9Ls. Whether the variations are responsible for the functional differentiation of OsYB7L and OsYB9L needs to be further elucidated.

In terms of the NF-YC family, OsNF-YC8, OsNF-YC9, OsNF-YC10, OsNF-YC11, and OsNF-YC12 were phylogenetically distinct from the other rice NF-YCs (Supplementary Fig. S2C, F). The intact nucleotide sequences of *OsNF-YC8*, *OsNF-YC9*, and *OsNF-YC10* were very similar (Supplementary Fig. S5A), indicating that these genes were formed by recent duplication events. Likewise, OsNF-YC11 and OsNF-Y12 showed high similarities (Supplementary Fig. S5B). Because of this, we used the core sequences of OsNF-YC8 and OsNF-YC12 to represent OsNF-YC8,9,10 and OsNF-YC11,12, respectively, to identify their homologs in other plant species. The results showed that close homologs of OsNF-YC8 and OsNF-YC12 were exclusively present in monocots but not in dicots (Fig. 3B and Supplementary Fig. S6), which clearly suggests that this group of genes evolved after the divergence of dicots and monocots. Together, our results showed that the endosperm-preferentially expressed NF-YA, NF-YBs, and NF-YCs have unique sequence features that separate them from widely expressed canonical NF-Ys.

### Endosperm-preferential expression of NF-Ys in other monocot species

Zm00001d017899 and Sb04g029340 were the most similar homologs of OsNF-YB1 in maize and sorghum, respectively (Fig. 3A). By searching RNA-seq data deposited in the Expression Atlas database, we found that these genes are predominantly or exclusively expressed in endosperm (Supplementary Fig. S7A). Likewise, Zm00001d045772 (GRMZM2G167576) and Sb10g010520, the close homologs of *OsNF-YB9* (Fig. 3A), are also exclusively expressed in endosperm (Supplementary Fig. S7B). Sb08g006350, Sb08g003700, and Sb07g004410 are sorghum homologs of the endosperm-preferential OsNF-YCs (Fig. 3B). Sb08g006350 and Sb07g004410 were only detectable in endosperm (Supplementary Fig. S7C), but the expression of Sb08g003700 is not available in the Expression Atlas. The maize genome encodes only one homology gene, Zm00001d023466 (GRMZM2G052499), of *OsNF-YC8* or *OsNF-YC12*. Although the Expression Atlas had no information about this gene, microarray-based experiments indicated that Zm00001d023466 was predominantly expressed in endosperm (Supplementary Fig. S7E). Taken together, these results clearly indicate that monocots have evolved a group of phylogenetically conserved NF-YBs and NF-YCs that are predominantly expressed in endosperm.

We also explored the expression of the *OsNF-YB7* homologs in other monocot species. Similar to *OsNF-YB7*, Zm00001d017898 (GRMZM2G011789) and Zm00001d051697 (GRMZM2G124663) of maize and Sb04g029350 of sorghum were predominantly expressed in the embryo (Supplementary Fig. S7D, F, G), indicating that these genes may function in seed development with a similar role to that of *LEC1* in Arabidopsis.

**Fig. 4.**
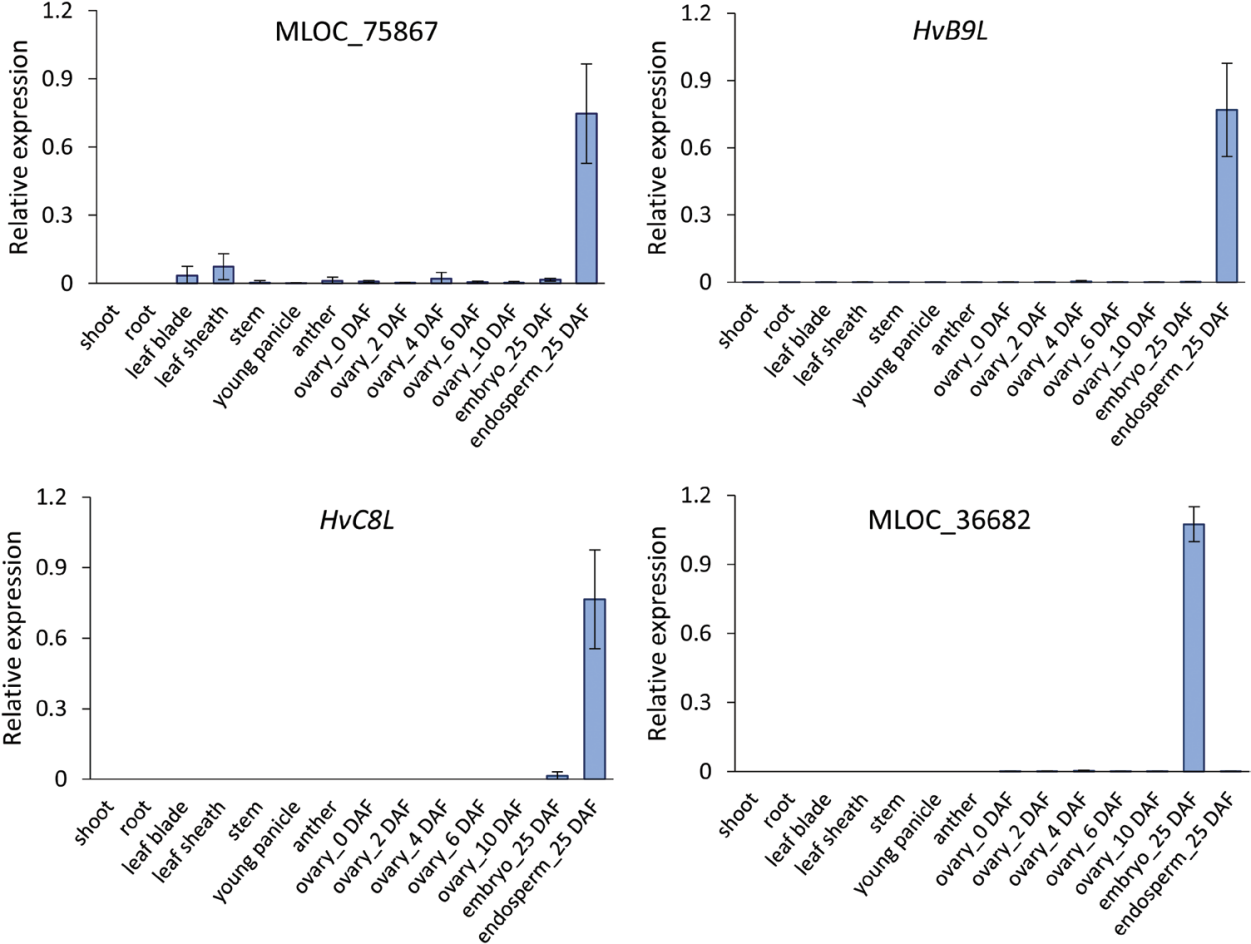
Gene expression of the barley homologs of caryopsis-preferential OsNF-Ys. Three biological replicates were used for qRT-PCR analysis; data are means (±SD). The tissues used for expression analysis are indicated. DAF, days after fertilization. MLOC_75867 is the barley homolog of OsNF-YB1, HvB9L is that of OsNF-YB9, HvC8L is that of OsNF-YC8, and MLOC_36682 is that of OsNF-YB7. (This figure is available in colour at *JXB* online.)

MLOC_75867 and MLOC_36682 were the barley homologs of OsNF-YB1 and OsNF-YB7, respectively (Fig. 3A). However, we failed to find the barley homologs of OsNF-YB9, OsNF-YC8, and OsNF-YC12 through blastp, possibly due to poor annotation of the barley genome. Therefore, we used the core protein sequences of OsNF-YB9, OsNF-YC8, and OsNF-YC12 to perform tblastn searches against the barley genomic DNA sequence. We were then able to identify two genes, referred to hereafter as *HvB9L* and *HvC8L*, that showed high similarities to OsNF-YB9 and OsNF-YC8/12 (Supplementary Fig. S8). Next, we analysed expression of these barley genes in various tissues by real-time PCR. As expected, MLOC_75867, *Hv9BL*, and *Hv8CL* were predominantly expressed in the endosperm, whereas MLOC_36682 (an *OsNF-YB7* homolog) showed high expression in the embryo but not in the endosperm (Fig. 4).

The sequence similarities and conserved expression patterns of these endosperm-preferential NF-Y groups in different monocot species strongly suggest that differentiation of these genes occurred in a common ancestor of rice, maize, sorghum, and barley. Notably, *OsNF-YB1* (LOC_Os02g49410) and *OsNF-YB7* (LOC_Os02g49370) and their corresponding homology genes in maize (Zm00001d017899 and Zm00001d017898) and sorghum (Sb04g029340 and Sb04g029350) are all located on adjacent chromosomal regions (Supplementary Fig. S9), which further confirms that the gene duplication event responsible for the evolution of NF-YB1 and NF-YB7 was very ancient.

### Interactions between the endosperm-preferential OsNF-Ys

**Fig. 5.**
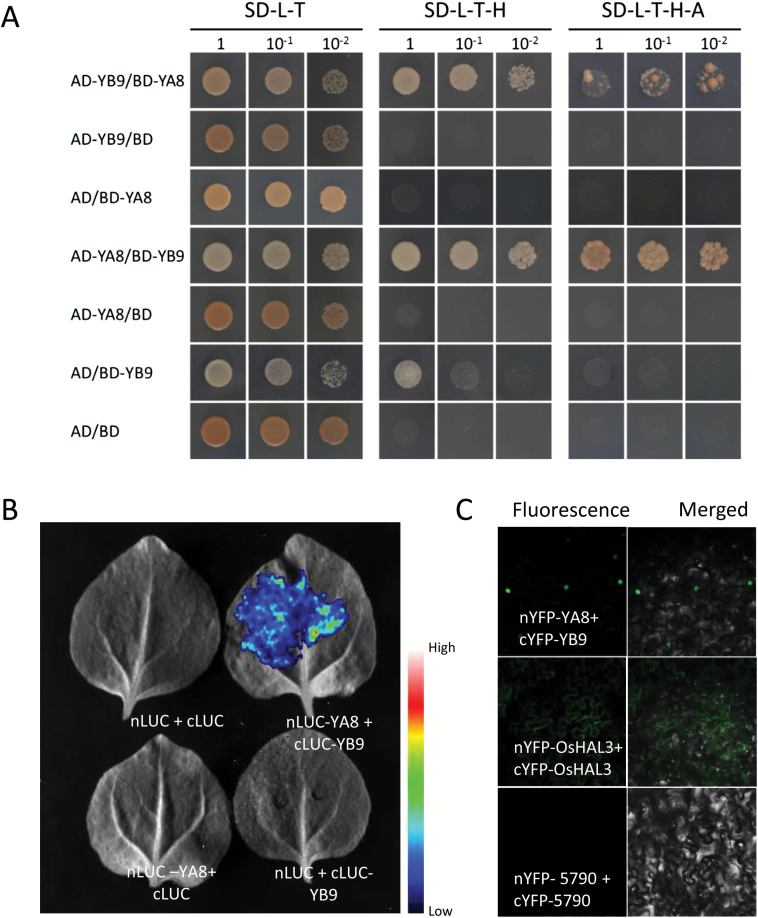
Interactions between OsNF-YA8 and OsNF-YB9. (A) Yeast-two-hybrid assays show interactions between OsNF-YA8 and OsNF-YB9. AD and BD indicate the activation domain and the DNA-binding domain of GAL4, respectively; AD-YA8 indicates the AD::OsNF-YA8 fusion, AD-YB9 indicates AD::OsNF-YB9, BD-YA8 indicates BD::OsNF-YA8, and BD-YB9 indicates BD::OsNF-YB9. Serial dilutions of the yeast cells expressing the indicated proteins were plated on non-selective (SD–L–T) or selective (SD–L–T–H and SD–L–T–H–A) medium. (B) Split luciferase complementation assays confirming the interaction between OsNF-YA8 and OsNF-YB9. nLUC indicates the N-terminal of luciferase, cLUC is the C-terminal of luciferase, nLUC-YA8 is the nLUC::OsNF-YA8 fusion, and cLUC-YB9 is the cLUC::OsNF-YB9 fusion. Different construct combinations were transiently co-expressed in tobacco epidermal cells. (C) BiFC assays show interactions between OsNF-YA8 and OsNF-YB9. OsNF-YA8 and OsNF-YB9 were fused with the N-terminal of YFP (nYFP) and the C-terminal of YFP (cYFP), respectively. OsHAL3, which can dimerize in the cytoplasm, was used as the positive control, while the gene LOC_Os01g05790 (5790), which cannot form a homodimer, was used as the negative control.

The core sequences of rice endosperm-preferential NF-Ys were somewhat different from the canonical NF-Ys (Supplementary Fig. S2); however, the homology modeling analysis showed no change of the protein structures in spite of sequence variabilities (Supplementary Fig. S10). The results indicated that these endosperm-preferential NF-Ys might interact with different members as the canonical NF-Ys. To test this idea, we performed a yeast two-hybrid assay to detect protein interactions between different groups of endosperm-preferential OsNF-Ys. The NF-YBs, including OsNF-YB1, OsNF-YB9, and OsNF-YB7, were able to interact with any of the endosperm-preferential NF-YC family members (Supplementary Fig. S11). However, OsNF-YA8 was only able to interact with OsNF-YB9 (Fig. 5A and Supplementary Fig. S12). Despite the high similarities between OsNF-YB7 and OsNF-YB9, OsNF-YA8 did not show interactions with OsNF-YB7 in yeast (Supplementary Fig. S12).

**Fig. 6.**
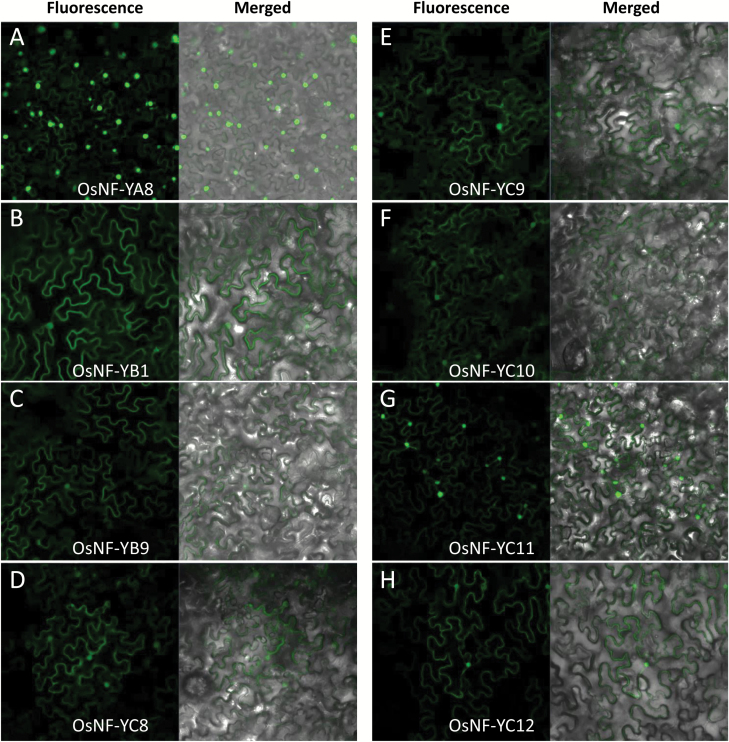
Subcellular localization of the endosperm-preferential NF-Ys of rice in tobacco epidermal cells. The florescence signal of the OsNF-YA8::Venus fusion was predominantly expressed in nucleus (A) while the other OsNF-Y::Venus fusions were expressed in the cytoplasm as well as in nucleus (B–H). The NF-Ys were N-terminally fused to a Venus tag.

Usually, NF-YA does not interact with the NF-YB or NF-YC monomers (Hackenberg *et al.*, 2012). To confirm the interaction between OsNF-YA8 and OsNF-YB9, we conducted a split luciferase complementation assay in tobacco epidermal cells. After co-filtration of nLUC (the N-terminal of luciferase) and cLUC (the C-terminal of luciferase), we could not detect luciferase activity (Fig. 5B). However, co-expression of nLCU-OsNF-YA8 and cLUC- OsNF-YB9 showed a high luciferase activity (Fig. 5B). In addition, neither the combination of nLCU-OsNF-YA8 and cLUC, nor that of nLCU and cLUC-OsNF-YB9 could activate luciferase activity (Fig. 5B). The results validated the interaction between OsNF-YA8 and OsNF-YB9 *in planta*. A BiFC assay also confirmed the interaction. The nYFP::OsNF-Y8A and cYFP::OsNF-YB9 constructs were co-expressed in tobacco epidermal cells. Fluorescence signals could be observed in the nucleus (Fig. 5C), which was in agreement with observations that both OsNF-YA8 and OsNF-YB9 were targeted in the nucleus (Fig. 6A, B).

### Subcellular localization of the endosperm-preferentially expressed OsNF-Ys

To detect the subcellular localization of the endosperm-preferential NF-Ys, we N-terminally fused the NF-Ys with a Venus-tag and transiently expressed the recombinant proteins in tobacco epidermal cells. The results indicated that NF-YA8 was predominately targeted to the nucleus (Fig. 6A), while the other endosperm-preferential OsNF-Ys were localized in both the cytoplasm and nucleus (Figs. 6B–H).

**Fig. 7.**
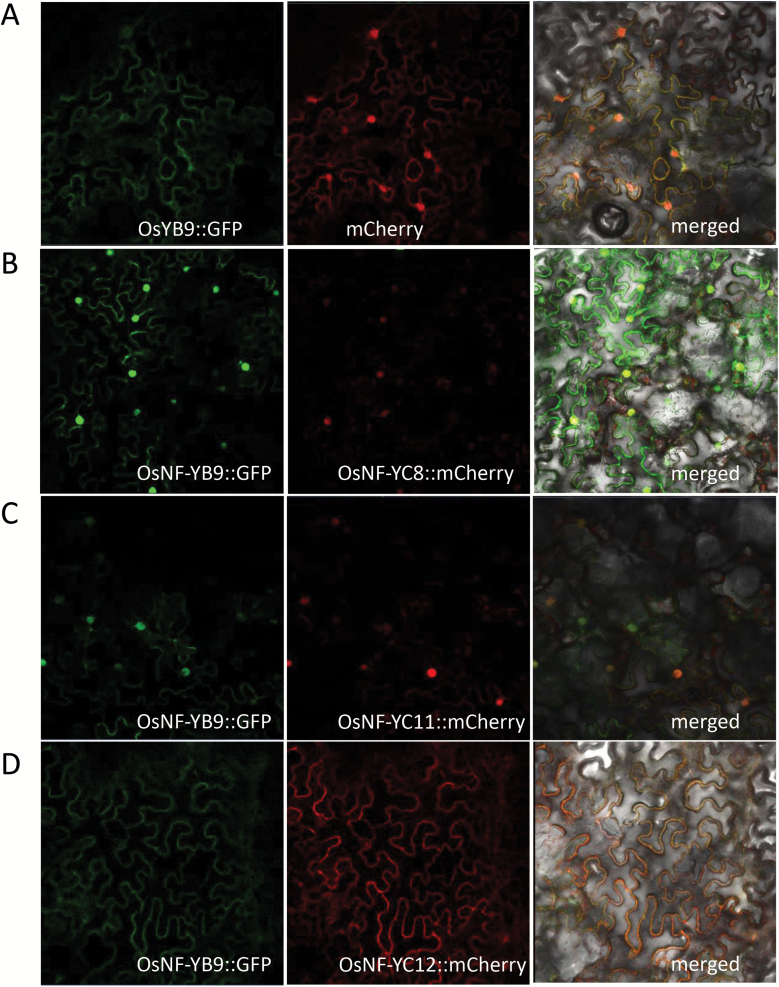
Co-expression of OsNF-YB9 and endosperm-preferential OsNF-YCs affected subcellular localization of the proteins. The fusion of OsNF-YB9::GFP was co-expressed with the mCherry empty vector (A), OsNF-YC8::mCherry (B), OsNF-YC11::mCherry (C), and OsNF-YC12::mCherry (D) in tobacco leaves. The mCherry tag was C-terminally fused with different OsNF-YCs.

Endosperm-preferential NF-YBs of rice were able to interact with NF-YCs in yeast (Supplementary Fig. S11). Previous studies suggested that a NF-YB and a NF-YC can form a heterodimer in the cytoplasm and then translocate to the nucleus (Laloum *et al.*, 2013). Therefore, we co-expressed OsNF-YB9::GFP with OsNF-YCs::mCherry in tobacco to investigate how the interactions affected their localization. The results showed that co-expression of OsNF-YB9::GFP and the mCherry empty vector did not change the subcellular localization of OsNF-YB9. The GFP signal could be observed in the cytoplasm as well as in the nucleus (Fig. 7A). However, when OsNF-YB9::GFP was co-expressed with OsNF-YC8::mCherry or OsNF-YC11::mCherry, the GFP signal was predominantly localized in the nucleus (Fig. 7B, C). The results suggested that interactions between OsNF-YB9 and OsNF-YC8 or OsNF-YC11 in the cytoplasm might facilitate their transportation to the nucleus. Notably, co-expression of OsNF-YB9 and OsNF-YC12 acted to retain the proteins in the cytoplasm rather than promoting their targeting to the nucleus (Fig. 7D).

### Endosperm preferential NF-YBs can interact with ERFs

**Fig. 8.**
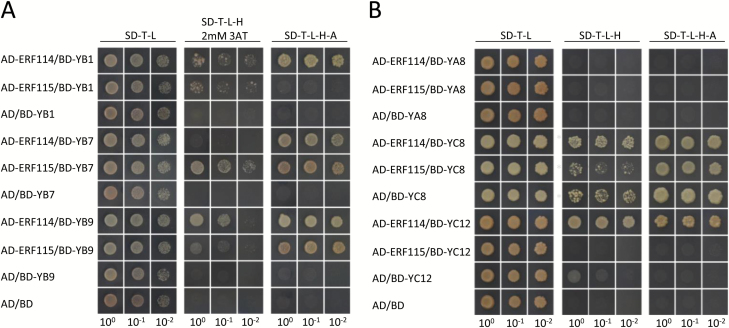
Interactions between seed-preferential OsNF-Ys and ERFs. (A) OsNF-YB1, OsNF-YB7, and OsNF-YB9 interacted with OsERF114 or OsERF115. BD-YB1, BD-YB9, and BD-YB7 indicate respectively that the OsNF-YB1, OsNF-YB9, and OsNF-YB7 were C-terminally fused with the DNA-binding domain (BD) of GAL4. AD-ERF114 and AD-ERF115 indicate that the OsERF114 and OsERF115 were respectively C-terminally fused with the activation domain (AD) of GAL4. Serial dilutions of the yeast cells expressing the indicated proteins were plated on non-selective (SD–L–T) or selective (SD–L–T–H with 2 mM 3AT or SD–L–T–H–A) medium. (B) OsNF-YC12 interacted with OsERF114 in yeast. BD-YA8, BD-YC8, and BD-YC12 indicate that OsNF-YA8, OsNF-YC8, and OsNF-YC12 were respectively C-terminally fused with the BD. Serial dilutions of the yeast cells expressing the indicated proteins were plated on non-selective (SD–L–T) or selective (SD–L–T–H or SD–L–T–H–A) medium. High self-activation activity of OsNF-YC8 was observed: yeast cells expressing the AD and the BD::OsNF-YC12 fusions survived on the selective (SD–L–T–H with 5 mM 3AT or SD–L–T–H–A) medium. (This figure is available in colour at *JXB* online.)

A previous study showed that OsNF-YB1 can interact with an ERF protein, OsERF115, to form NF-YB/NF-YC/ERF heterotrimers in the nuclei of rice endosperm cells (Xu *et al.*, 2016). We performed yeast-two-hybrid assays to test whether the other NF-YBs have the same ability. The results suggested that OsNF-YB9 was able to interact with OsERF114 and OsERF115 (Fig. 8A), but not with OsERF74 and OsERF72 (Supplementary Fig. S13). Notably, OsNF-YB7 also showed interactions with OsERF115, and possibly with OsERF114 as well (Fig. 8A).

We also investigated potential interactions between OsERF114/115 and the endosperm-preferential NF-YA and NF-YC family members. None of the ERFs interacted with OsNF-YA8 (Fig. 8B); however, OsNF-YC12 could interact with OsERF114 in yeast (Fig. 8B). Due to the self-activation activity of OsNF-YC8 (Fig. 8B), the interactions between OsERFs and OsNF-YC8 need to be further confirmed. Interestingly, the expression patterns of *OsERF114* and *OsERF115* were very similar to those of the endosperm-preferential NF-Ys (Supplementary Fig. S14), suggesting that OsERF114 and OsERF115 may co-ordinate with endosperm-preferential NF-YB and NF-YC members to regulate rice endosperm development.

### Transcriptional activation activity

**Fig. 9.**
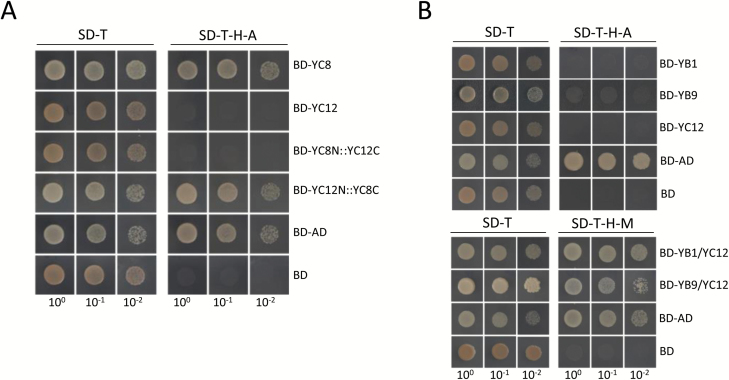
Transcriptional activation activities of the endosperm-preferential OsNF-Ys. (A) The C-terminal of OsNF-YC8 is important for the transcriptional activation activity. BD and AD indicate the DNA-binding domain and activation domain of GAL4, respectively. The serially diluted yeast cells expressing the BD::OsNF-YC8 fusion (BD-YC8) and the BD::OsNF-YC12 fusion (BD-YC12) could grow on the non-selective medium (SD–T), but only the transformants expressing BD-YC8 survived on the selective medium (SD–T–H–A), similar to how the positive control BD::AD-expressing yeast cells performed. When the C-terminal of OsNF-YC12 (amino acid residuals 361–984) was used to replace the C-terminal of OsNF-YC8 (385–1392), the fusion (BD-YC8N::YC12C) lost transcriptional activation activity. In contrast, the OsNF-YC12 N-terminal (1–360) and OsNF-YC8 C-terminal (385–1392) fusion (BD-YC12N::YC8C) showed transcriptional activation activity. (B) The OsNF-YB1 and OsNF-YC12 dimer showed transcriptional activation activity in yeast. The BD::OsNF-YB1, BD::OsNF-YB9, and BD::OsNF-YC12 fusions did not show transcriptional activation activity; but yeast cells simultaneously expressing BD::OsNF-YB1 and OsNF-YC12 (BD-YB1/YC12), and BD::OsNF-YB9 and OsNF-YC12 (BD-YB9/YC12) could survive on the selective medium (SD–H–T–M). The OsNF-Ys were cloned into pGBKT7 (top) and pBridge (below). (This figure is available in colour at *JXB* online.)

It has not yet been determined whether the members of NF-Y family have transcriptional activation activity in plants. Therefore, we fused the endosperm-preferential NF-Ys of rice with the GAL4 binding domain to detect their transcriptional activation ability in yeast. The OsNF-YA8, OsNF-YB1, OsNF-YB9, and OsNF-Y7B fusions were not able to survive on the selective synthetic drop-out medium (Supplementary Fig. S15), which suggested that these proteins alone are not capable of transcription activation. The OsNF-YC8, OsNF-YC9, and OsNF-YC10 fusions could survive on the drop-out medium like the positive control (a fusion of the GAL4 activation domain and the GAL4 DNA-binding domain), whereas the OsNF-YC11 and OsNF-YC12 fusions did not show any transcriptional activation activities (Supplementary Fig. S15). Next, we fused the N-terminal (containing the core domain of NF-YC) of OsNF-YC8 with the C-terminal of OsNF-YC12 (designed as YC8N::YC12C hereafter), and found that this chimeric protein did not have transcriptional activity (Fig. 9A). In contrast, transformants with the fusions of the N-terminal of OsNF-YC12 and the C-terminal of OsNF-YC8 (YC12N::YC8C) could survive on the selective medium (Fig. 9A), which indicated that the C-terminal of OsNF-YC8 determines the transcriptional activation activity.

Because it was lacking transcriptional activation activity in OsNF-YC11 and OsNF-YC12 alone, we assumed that the transcriptional activation ability of these NF-YC family members might require the assistance of the NF-YBs with which they interact. OsNF-YB1 and OsNF-YC12 could interact with each other (Supplemental Fig. S11A), but neither OsNF-YB1 nor OsNF-YC12 showed transcriptional activation activity (Fig. 9B and Supplementary Fig. S15). However, when we co-expressed OsNF-YC12 and the fusions of OsNF-YB1 and the GAL4 DNA-binding domain in yeast, the transformants could survive on the selective drop-out medium (Fig. 9B). The results strongly supported our hypothesis that the dimer formed by OsNF-YC12 and OsNF-YB1 has transcriptional activation activity, even though each of the proteins alone does not have that function.

### Phenotypic analysis of the *OsNF-Ys* knockout mutants

**Fig. 10.**
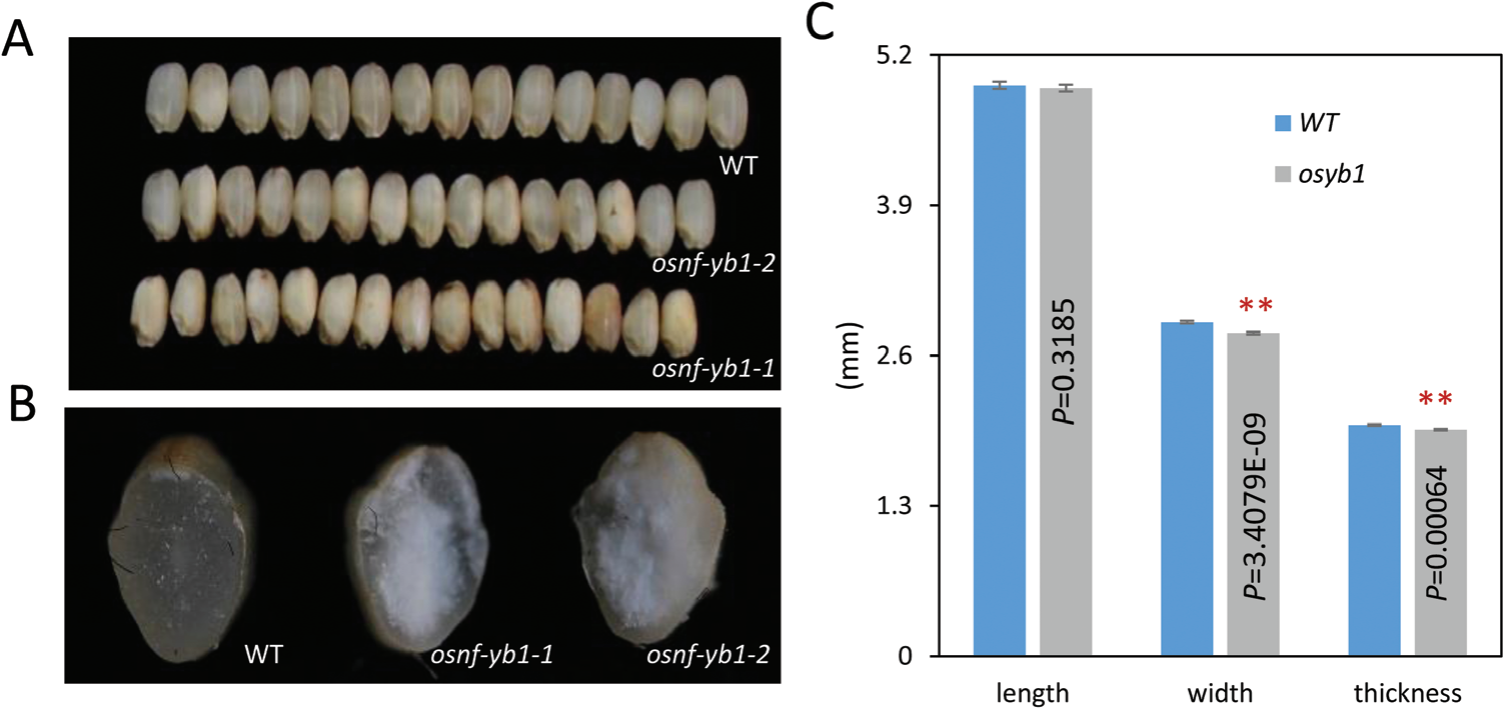
Seed morphologies of *OsNF-YB1* mutants generated using the CRISPR/Cas9 approach. (A) Seed morphologies of the *osnf-yb1* mutants and the wild-type (WT): *osnf-yb1-1* and *osnf-yb1-2* denote two different mutant alleles. (B) Floury endosperm produced by the *osnf-yb1* mutants. (C) Size of brown seed of the *osnf-yb1-1* mutant compared with the wild-type. **P<0.01; *t*-test (*n*=100).

To examine the biological roles of the endosperm-preferential *OsNF-Y*s in seed development, knockout mutants of these genes were generated using the CRISPR/Cas9 approach. We obtained homozygous mutants of *OsNF-YB1*, *OsNF-YC8*, *OsNF-YC11*, and *OsNF-YC12*, and found that they did not show any visible defects in vegetative development, which was in agreement with their endosperm-preferential expression pattern. Consistent with previous findings (Sun *et al.*, 2014; Bai *et al.*, 2015; Xu *et al.*, 2016), the *osnf-yb1* mutant showed reduced seed size and increased chalkiness of the endosperm (Fig. 10). However, homozygous mutants of *osnf-yc8*, *osnf-yc11*, and *osnf-yc12* did not display obvious abnormalities in terms of seed size and endosperm appearance (Supplementary Fig. S16). This may be due to redundancies among *OsNF-YC8*, *OsNF-YC9*, *OsNF-YC10*, *OsNF-YC11*, and *OsNF-YC12*.

## Discussion

Functional analyses have shown that the NF-Ys of plants may play multiple roles in stress response, flowering time regulation, embryo development, and chloroplast biogenesis (see reviews by Petroni *et al.*, 2012; Laloum *et al.*, 2013). However, the function of plant NF-Ys with regards to endosperm development is largely unknown. To explore this, we conducted a comprehensive phylogenetic analysis of the endosperm-preferential OsNF-Ys. Our results suggested that there are phylogenetically conserved and monocot-specific NF-YB and NF-YC groups that probably function in cereal endosperm development. We infer that the genes evolved after the divergence of dicots and monocots, because dicots lack close homologs of these genes (Supplementary Figs 3, 4, 6). Previous studies have shown that LEC1-like NF-YBs are essential for seed maturation and embryo development (Kwong *et al.*, 2003; Lee *et al.*, 2003). Interestingly, we found that the LEC1-like family could be further divided into two subgroups (Supplementary Fig. S4). The OsYB7Ls are phylogenetically closer to LEC1, whereas the OsYB9Ls are monocot-specific. The *OsYB7L*s are usually expressed in the embryo, whereas the *OsYB9L*s are predominantly expressed in the endosperm (Figs 2, 4 and Supplementary Fig. S7). The distinct expression patterns suggested that the LEC1-like family members OsYB7Ls and OsYB9Ls have been sub-functionalized for monocot caryopsis development.

A systematic analysis of different caryopsis-preferential NF-Ys showed that OsNF-YA8 could interact with OsNF-YB9 (Fig. 5 and Supplementary Fig. S12). This was surprising, because NF-YAs usually bind to NF-YB/NF-YC dimers but not to any NF-YB or NF-YC monomers (Hackenberg *et al.*, 2012). The functional significance of this interaction requires further study. Notably, in spite of having high similarities to OsNF-YB9, OsNF-YB7 did not interact to OsNF-YA8 in yeast (Supplementary Fig. S12). This raises the possibility that the sequence outside the core domain may contribute to the interactions between OsNF-YA8 and OsNF-YB9. In Arabidopsis, NF-YA family members showed variability in CCAAT-binding activity (Calvenzani *et al.*, 2012). Our phylogenic analysis suggested that OsNF-YA8 is a recently evolved gene as it only can be found in the *Oryza* genus, and it carries many variations within the core domain compared with the canonical NF-YA family members (Supplementary Fig. S2A). Several conserved residuals are differentiated in the A2 domain of OsNF-YA8, and this domain is involved in recognition and binding to the CCAAT motif (Gnesutta *et al.*, 2017). It remains to be determined whether the sequence variations allow OsNF-YA8 to recognize *cis*-elements other than the CCAAT-box.

In addition to NF-YAs, NF-YB/NF-YC dimers may interact with other transcription factor family members to regulate the expression of downstream targets (Yamamoto *et al.*, 2009; Cao *et al.*, 2014; Huang *et al.*, 2015; Xu *et al.*, 2016). A previous study showed that OsNF-YB1 was able to interact with OsERF115 to modulate grain filling in rice (Xu *et al.*, 2016). In our present study, we found that OsNF-YB9 and OsNF-YB7 of rice could also interact with OsERF114 and OsERF115 (Fig. 8). Moreover, The NF-YC family member OsNF-YC12 showed interactions with OsERF114 as well. Similar to OsNF-Y genes, the expression of *OsERF114* and *OsERF115* was endosperm-specific (Supplementary Fig. S14), which suggests that the interactions between the ERFs and the NF-Y complexes are probably important for endosperm development. However, the physiological role of the interaction requires further investigation.

Our understanding of the transcriptional activation activity of plant NF-Ys is limited. Here, we found that OsNF-YC8, OsNF-YC9, and OsNF-YC10 had transcriptional activation activities but that OsNF-YC11 and OsNF-YC12 did not (Fig. 9 and Supplementary Fig. S15). However, OsNF-YC12 did show transcriptional activation activity when co-expressed with OsNF-YB1 or OsNF-YB9 in yeast (Fig. 9B). The results suggested that the co-ordination of NF-YB and NF-YC subunits probably confers the transcriptional activation activity of a NF-Y transcription factor complex. Previous studies in mammals have shown that the transcriptional activity of NF-Ys was dependent on the glutamine-rich domains that locate outside the core domains of the NF-YB and NF-YC subunits (Coustry *et al.*, 1995, 1996). Consistent with these findings, our results suggested that the C-terminal, but not the conserved domain of OsNF-YC8, was an essential requirement for its transactivational activity (Fig. 9A). In addition, the OsNF-YB/OsNF-YC dimers could recruit other transcription factors to form a more complicated complex to modulate the expression of downstream genes. As an example, rice is likely to form a complex that consists NF-Ys, Hd1, OsHAPL1, and the general transcription factors to control plant flowering (Zhu *et al.*, 2017). A NF-Y complex may also recruit transcription repressors to regulate gene expression (Li *et al.*, 2011). Leyva-González *et al.* (2012) showed that NF-YA2 of Arabidopsis could act like a repressor of a subset of genes that lack the CCAAT-box. Therefore, an alternative interpretation of the lack of transcriptional activation shown by OsNF-YA8, OsNF-YB1/7/9, or OsNF-YC11/12 alone in yeast could be because these NF-Ys function as transcriptional repressors rather than activators. These findings suggest that the function of NF-Ys in transcriptional regulation might be very divergent, and that the NF-Y complex of plants may require the co-ordination of multiple subunits for transcriptional activation activity.

**Fig. 11.**
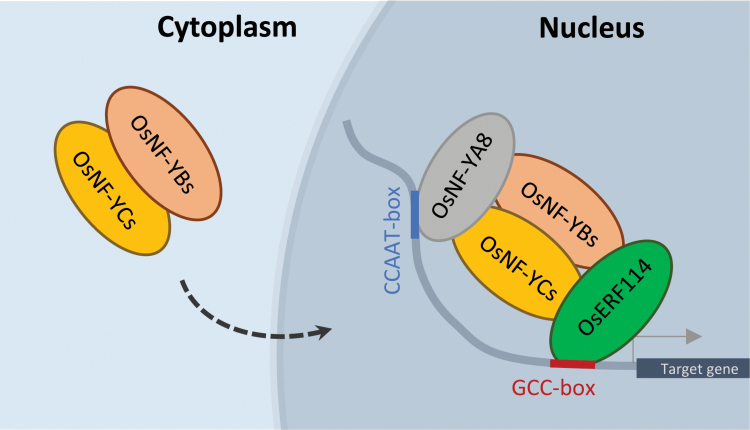
A hypothetical model showing the function of endosperm-preferential OsNF-Ys in rice endosperm development. The OsNF-YBs (OsNF-YB1/9 and possibly OsNF-YB7) and the OsNF-YCs (OsNF-YC8/9/10/11/12) are dimerized in the cytoplasm of endosperm cells and then imported into the nucleus to interact with OsNF-YA8 and OsERF114/115, or possibly with other transcription factors. OsNF-YA8 can recognize the CCAAT-motif while OsERF114/115 can bind to the GCC-box. The OsERFs co-ordinate with the OsNF-Y complex to regulate the downstream genes involved in endosperm development.

Previous studies and our results indicated that the physical interactions between an endosperm-preferential NF-YB and an endosperm-preferential NF-YC in the cytoplasm may facilitate their translocation to the nucleus, in which the NF-YB/NF-YC dimer can then bind with a NF-YA and/or some other factor, such as OsERF114, to assemble a functional transcription factor complex. In the complex, the NF-YA and ERF are responsible for CCAAT-box and GCC-box recognition, whereas the NF-YB and NF-YC contribute to transcriptional activation activity. The transcriptional factor complex can thus co-ordinately regulate the expression of downstream genes governing endosperm development (Fig. 11).

To further confirm that NF-Ys may play a role in endosperm development, we generated knockout mutants of OsNF-Ys. Possibly due to functional redundancy among OsNF-Ys, we did not observe any seed phenotype with the knockout lines of *osnf-yc8*, *osnf-yc11*, and *osnf-yc12* (Supplementary Fig. S16). Alternatively, there is a possibility that some of the genes are actually non-functional pseudogenes that have fortuitously retained an ability to interact, but that are expressed at relatively low levels and have no role in development. However, *osnf-yb1* displayed decreased seed size and increased chalkiness of the endosperm (Fig. 10). This was consistent with previous findings (Sun *et al.*, 2014; Bai *et al.*, 2015; Xu *et al.*, 2016) and strongly implies that some of the endosperm-preferential OsNF-Ys, if not all, are essential for rice endosperm development.

## Supplementary data

Supplementary data are available at *JXB* online.

Table S1. Primers used in the study.

Fig. S1. Expression of the rice endosperm-preferential NF-Ys in aleurone cells and endosperm.

Fig. S2. Amino acid sequence alignment and phylogenetic analysis of the conserved domains of rice NF-Ys.

Fig. S3. Neighbor-joining phylogenetic tree of the top 100 homologs of OsNF-YB1 and the NF-YB members of rice.

Fig. S4. Phylogenetic and alignment analysis of OsNF-YB7, OsNF-YB9, and their homologs.

Fig. S5. Multiple sequence alignment of the endosperm-preferential OsNF-YCs.

Fig. S6. Neighbor-joining phylogenetic tree of the top 100 homologs of OsNF-YC8 and the NF-YB members of rice.

Fig. S7. Gene expression of the homologs of rice endosperm-preferential NF-YCs in maize and sorghum.

Fig. S8. Multiple sequence alignments of OsNF-YB9 and HvB9L and of OsNF-YC8, OsNF-YC12, and HvC8L.

Fig. S9. Diagram showing the physical linkage of the *OsNF-YB7-like* and *OsNF-YB1-like* genes in rice, maize, and sorghum.

Fig. S10. Predicted structures of the conserved domains of NF-Ys.

Fig. S11. Yeast two-hybrid assays showing that OsNF-YB1, OsNF-YB7, and OsNF-YB9 interact with all the endosperm-preferential OsNF-YCs.

Fig. S12. Yeast two-hybrid assays showing that OsNF-YA8 interacts with OsNF-YB9, but not with other endosperm-preferential OsNF-Ys.

Fig. S13. Yeast two-hybrid assays showing that OsNF-YB1, OsNF-YB7, and OsYB9 do not show interactions with OsERF72 or OsERF74.

Fig. S14. *OsERF114* and *OsERF115* are activated after fertilization.

Fig. S15. Yeast two-hybrid assays showing that OsNF-YC8, OsNF-YC9, and OsNF-YC10 exhibit transcriptional activation activities.

Fig. S16. Seed morphologies of the seed-preferential *OsNF-Y*s mutants generated using the CRISPR/Cas9 approach.

Supplementary Table S1

Supplementary Figures S1-S16
