# Supplementary material for: A group of nuclear factor Y transcription factors are sub-functionalized during endosperm development in monocots
Source: J Exp Bot. 2018 Mar 5;69(10):2495–510. doi: 10.1093/jxb/ery087 (PMC5920288; doi:10.1093/jxb/ery087)
Supplement: Supplementary Figures S1-S16 [file ery087_suppl_supplementary_figures_s1-s16.pdf]

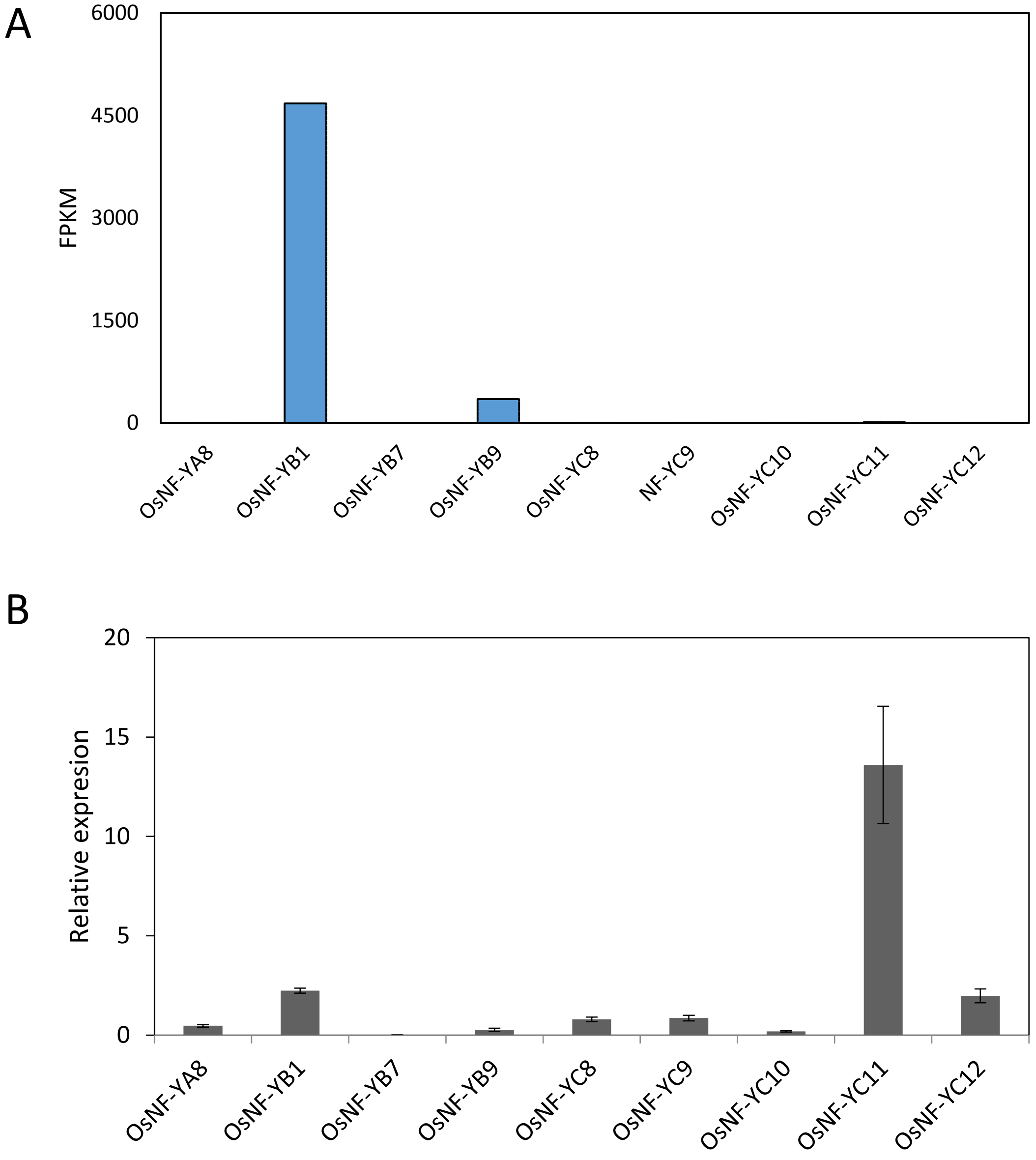

Supplementary Figure 1. Expression of the rice endosperm-preferential NF-Ys in aleurone cells (A) and endosperm (B). The RNA-seq data of (A) was obtained from GEO (GSE72185). 8 DAF endosperm of the *japonica* vatiety ZH11 (*O.sativa*) was used for laser capture microdissections. The same age endosperm of Kitaake was used for the real-time PCR assay of (B).

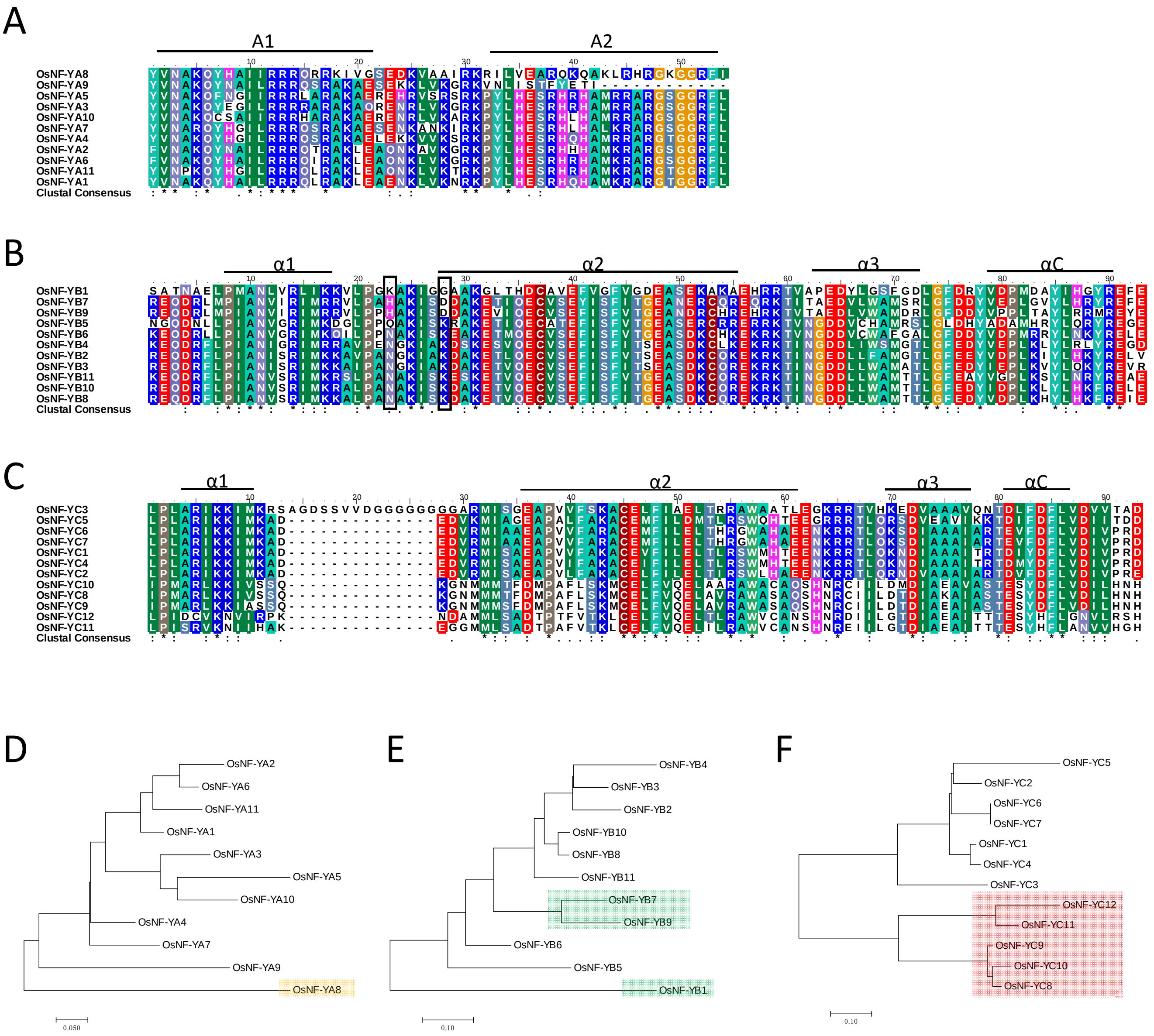

Supplementary Figure 2. Amino acid sequence alignment and phylogenetically analysis of the conserved domains of rice NF-Ys.

(A-C) Multiple alignments of the conserved domains of rice NF-YAs **(A)**, NF-YBs **(B)** and NF-YCs **(C)**. The conserved motifs are indicated on the top of alignments. A1 mediated the NF-YB and NF-YC interactions; A2 is involved in CCAAT binding. The diagnostic Asp (D) and His (H) of LEC1-like proteins are boxed in **(B)**. **(D-F)** Neighbor-joint trees of the rice NF-YAs **(D)**, NF-YBs **(E)** and NF-YCs **(F)**. The endosperm-preferential OsNF-Ys are highlighted.

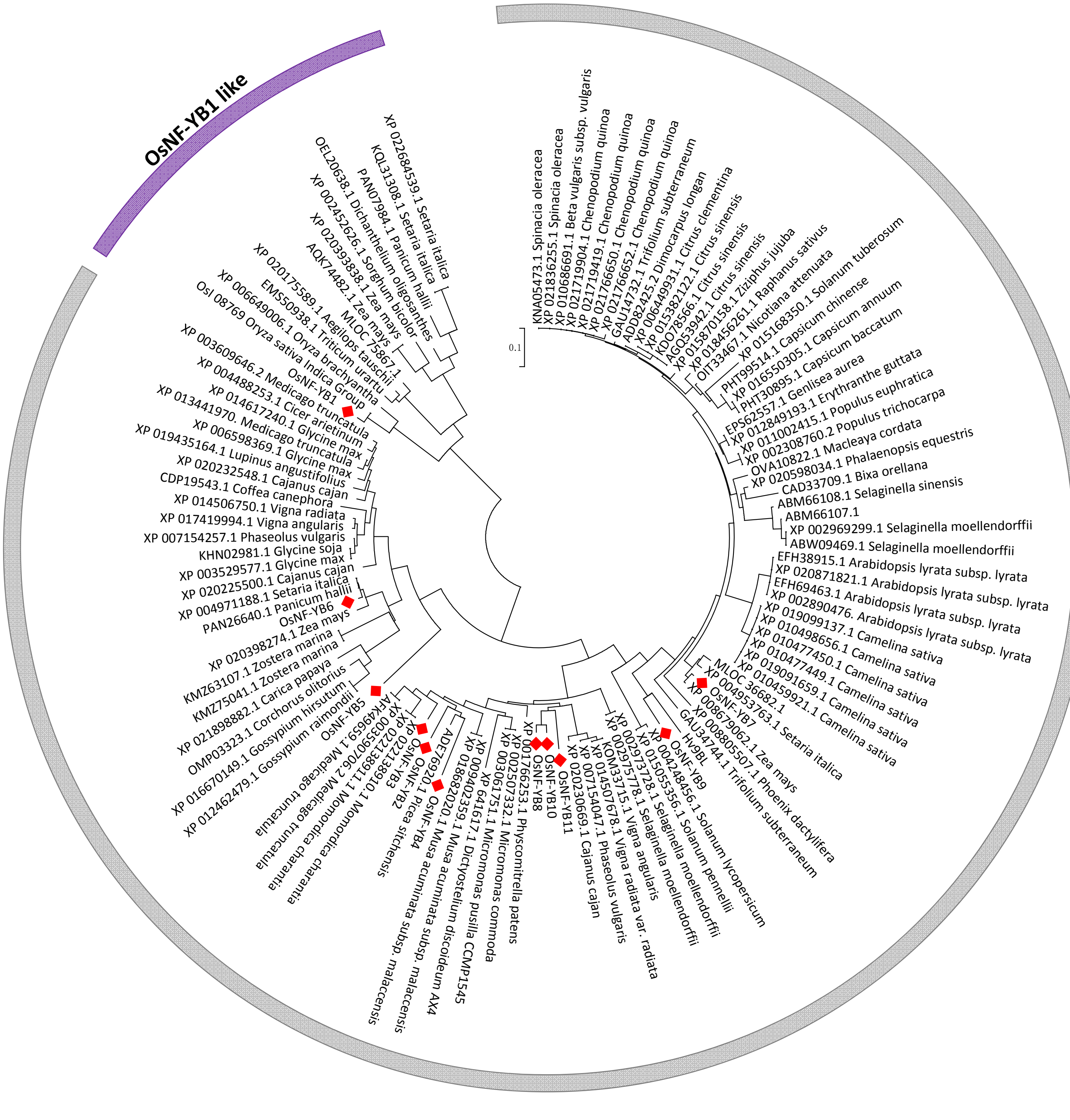

Supplementary Figure 3. Neighbor-joining phylogenetic tree of the top-100 homologs of OsNF-YB1 and the NF-YB members of rice. OsNF-YBs were indicated by red diamonds. The core sequence of OsNF-YB1 was used as the query for NCBI balstp search. The full protein sequences were used for tree construction. Species name were gave after the NCBI accession number of each protein.

A

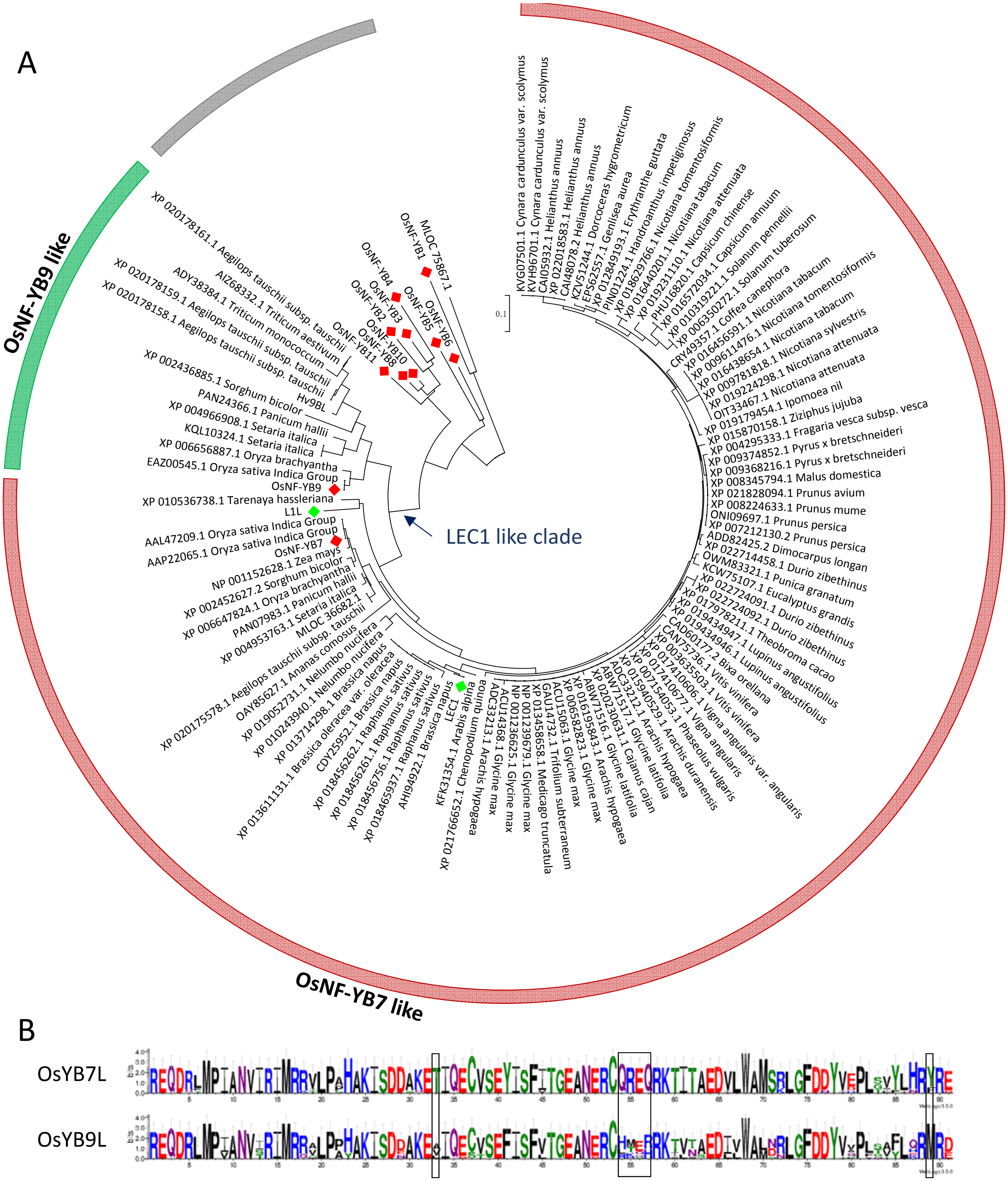

B

Supplementary Figure 4. Phylogenetic and alignment analysis of OsNF-YB7, OsNF-YB9 and their homologs. (A) Neighbor-joining phylogenetic tree of the top-100 homologs of OsNF-YB7 and the NF-YB members of rice. OsNF-YBs were indicated by red diamonds. LEC1 and L1L of *Arabidopsis* were indicated by green diamonds. The core sequence of OsNF-YB7 was used as the query for NCBI balstp search. The full protein sequences were used for tree construction. Species name were gave after the NCBI accession number of each protein. (B) Sequence logos of the conserved domains of OsNF-YB7 likes (OsYB7Ls) and OsYB9Ls. The green, purple, blue, red and black letters indicate polar, neutral, basic, acidic and hydrophobic amino acids, respectively. The overall height in each stack represents the conservation at the position, height of each residue letter indicates the relative frequency of the residue. The distinct sequences between OsYB7L and OsYB9L are boxed.

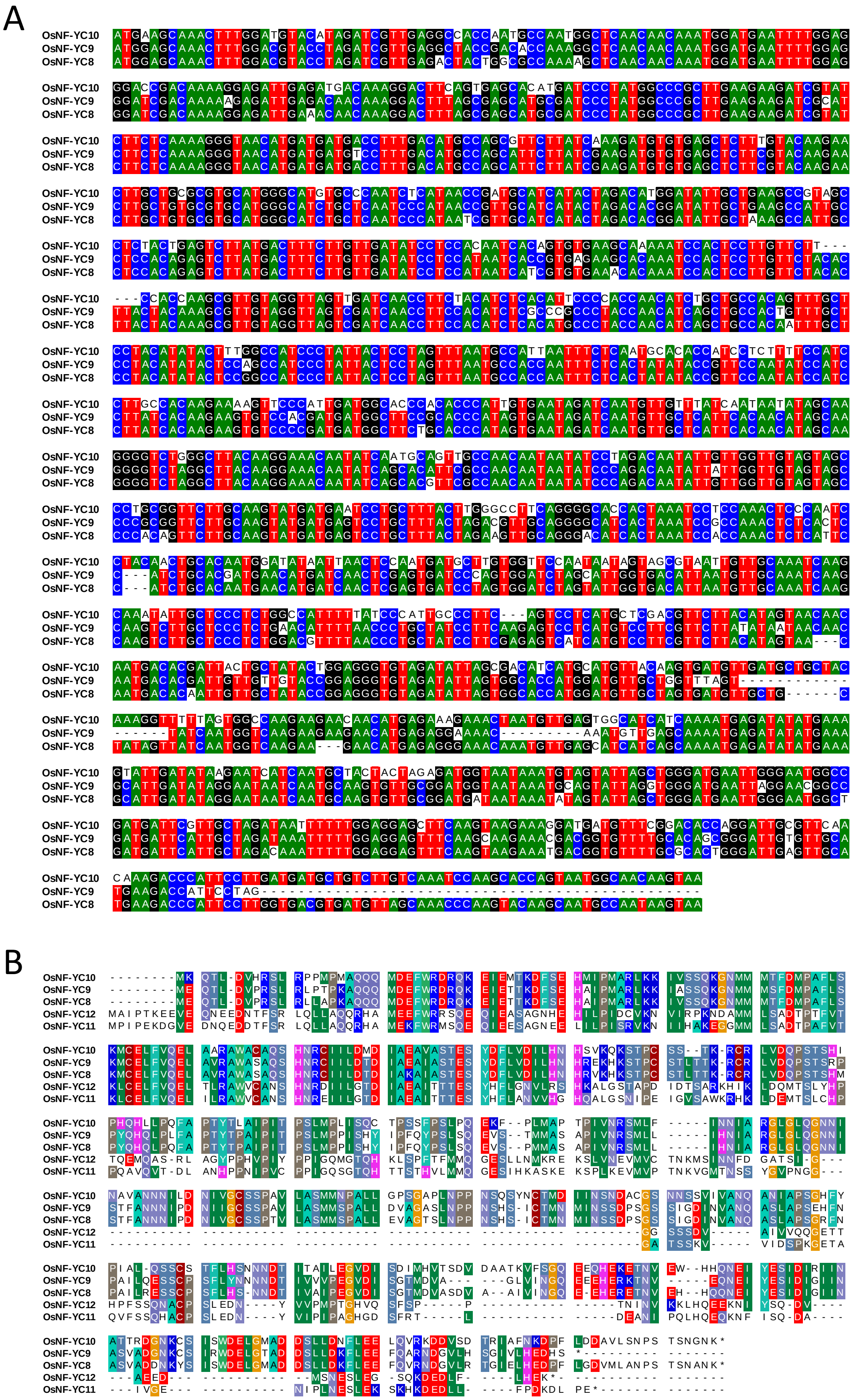

Supplementary Figure 5. Multiple sequence alignment of the endosperm-preferential OsNF-YCs. **(A)** DNA sequence alignment of OsNF-YC8/9/10. **(B)** Protein sequence alignment of OsNF-YC8/9/10/11/12.

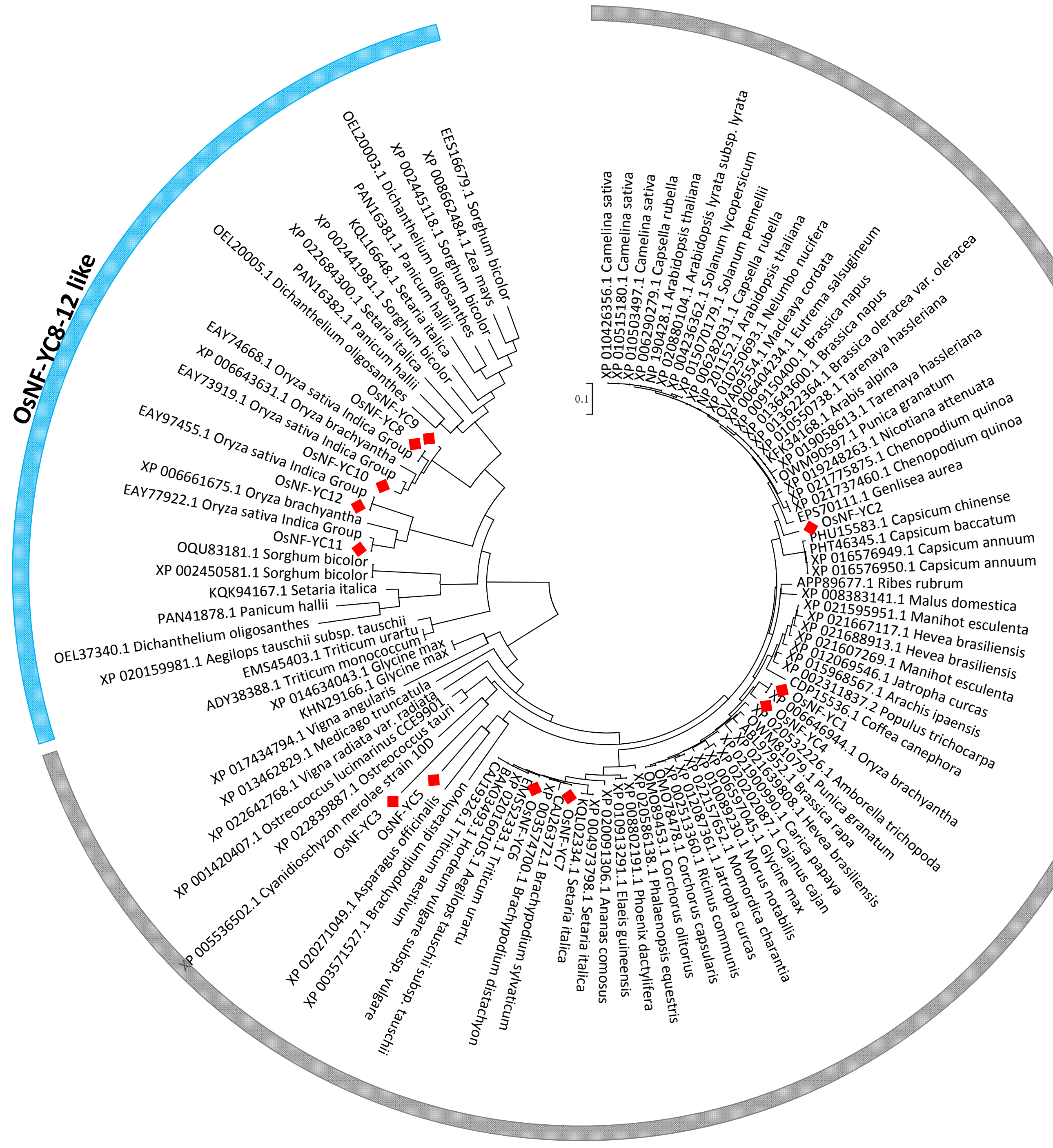

Supplementary Figure 6. Neighbor-joining phylogenetic tree of the top-100 homologs of OsNF-YC8 and the NF-YB members of rice. OsNF-YBs were indicated by red diamonds. The core sequence of OsNF-YC8 was used as the query for NCBI balstp search. The full protein sequences were used for tree construction. Species name were gave after the NCBI accession number of each protein.

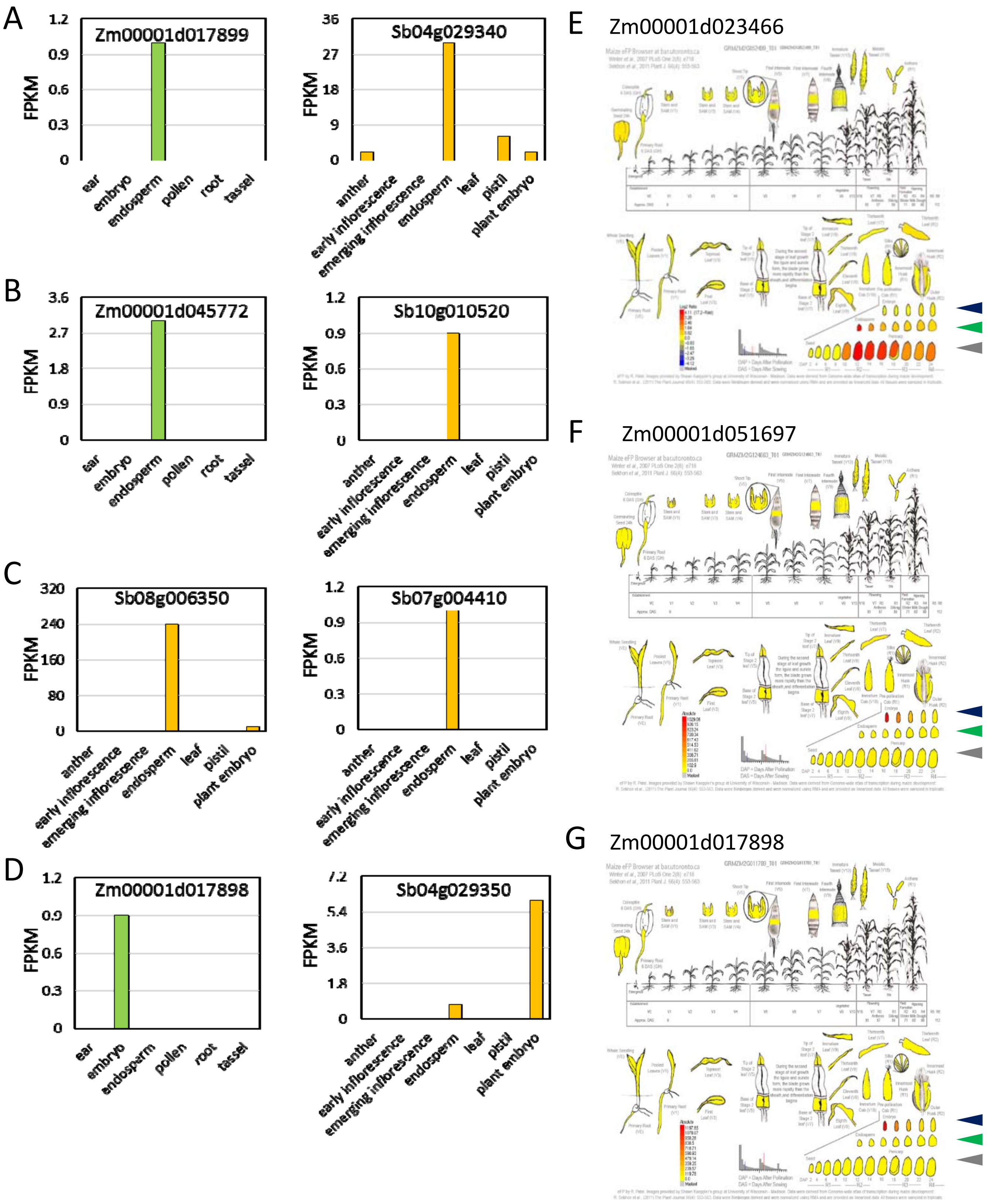

Supplementary Figure 7. Gene expression of the homologs of rice endosperm-preferential NF-YCs in maize and sorghum. The RNA-seq data of **(A-D)** was obtained from the Expression Atlas. The microarray data of **(E-G)** was obtained from the Sekhon et al Atlas deposited Maize eFP Browser. Zm00001d017899 and Sb04g029340 are homologs of OsNF-YB1; Zm00001d045772 and Sb10g010520 are homologs of OsNF-YB9; Sb08g006350, Sb07g004410 and Zm00001d023466 are homologs of OsNF-YC8 or OsNF-YC12; Zm00001d017898, Zm00001d051697 and Sb04g029350 are homologs of OsNF-YB7. The gray, blue and green triangles in **(E-G)** indicate caryopsis, embryo and endosperm, respectively.

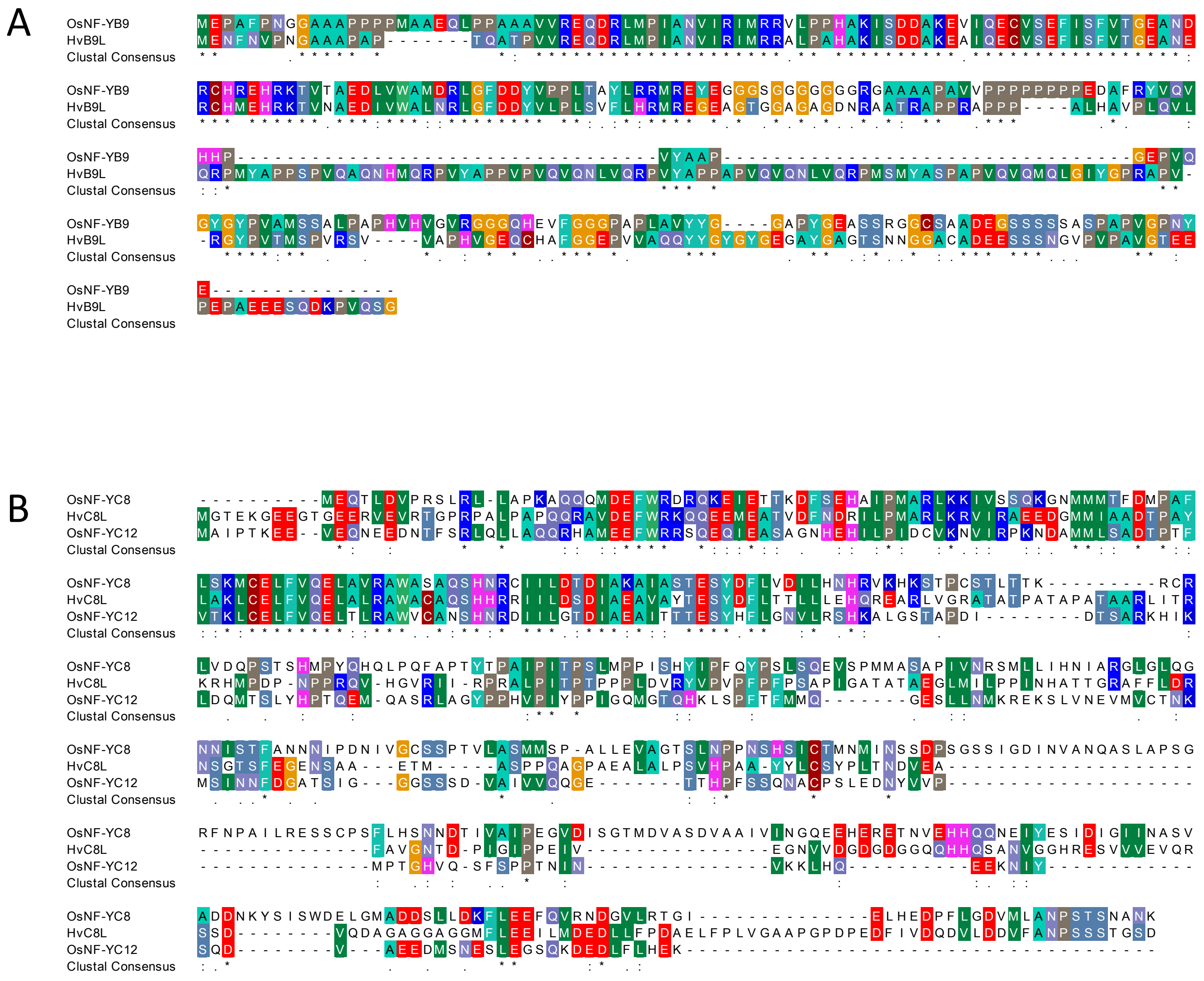

Supplementary Figure 8. Multiple sequence alignments of the OsNF-YB9 and HvB9L (A) and of the OsNF-YC8, OsNF-YC12 and HvC8L (B).

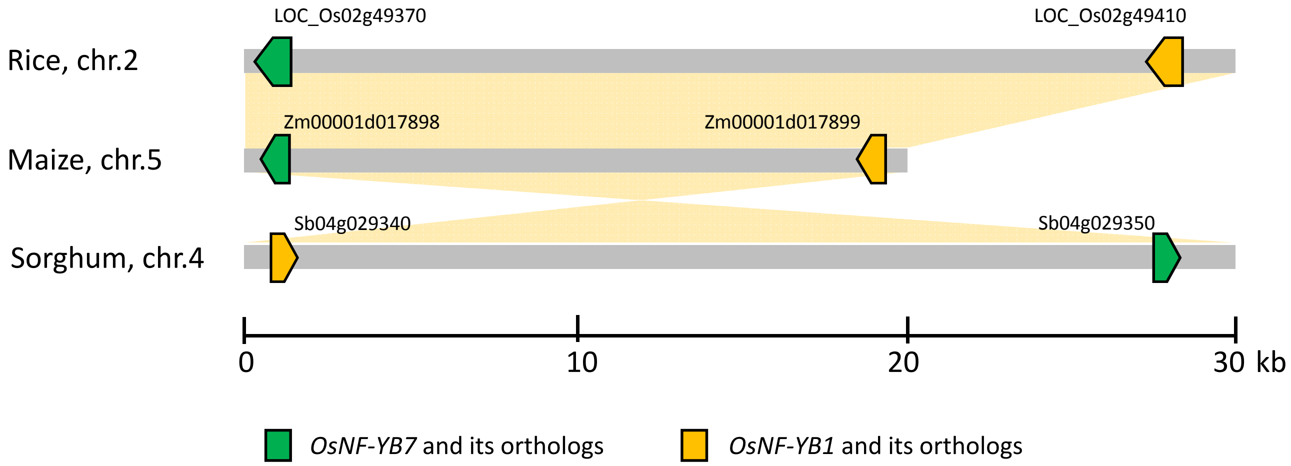

Supplementary Figure 9. Diagram showing the physical linkage of the *OsNF-YB7 like* and the *OsNF-YB1 like* genes in rice, maize and sorghum.
The gray bars indicate the chromosomes; the collinear regions are highlighted in yellow.

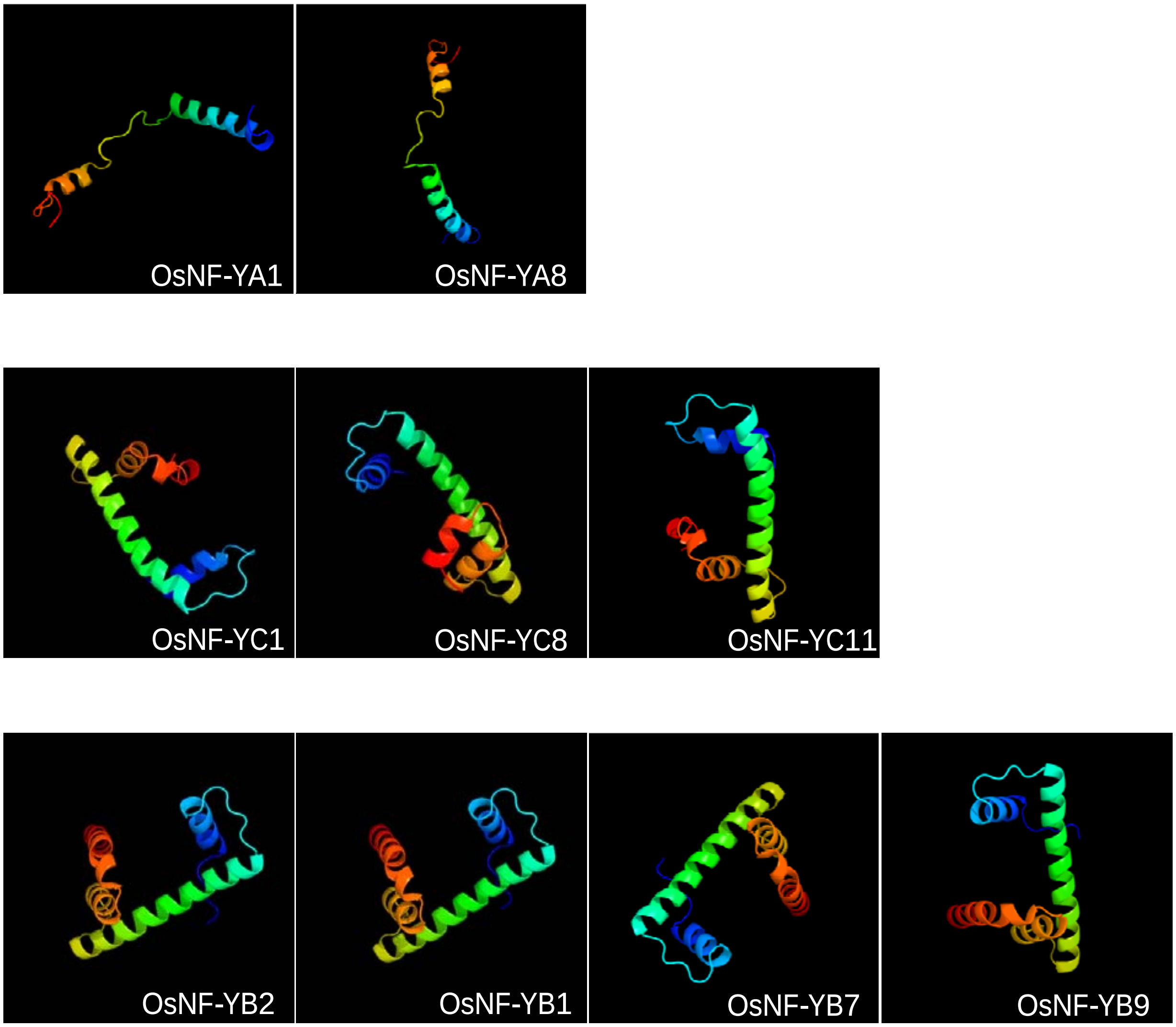

Supplementary Figure 10. Predicted structures of the NF-Ys' conserved domains.
OsNF-YA1, OsNF-YB2 and OsNF-YC1 are canonical NF-Ys which wildly expressed in rice. OsNF-YA8, OsNF-YB1/7/9 and OsNF-YC8/11 are endosperm-preferential NF-Ys of rice. The Intensive model of the Phyre2 web portal was used for NF-Ys modeling.

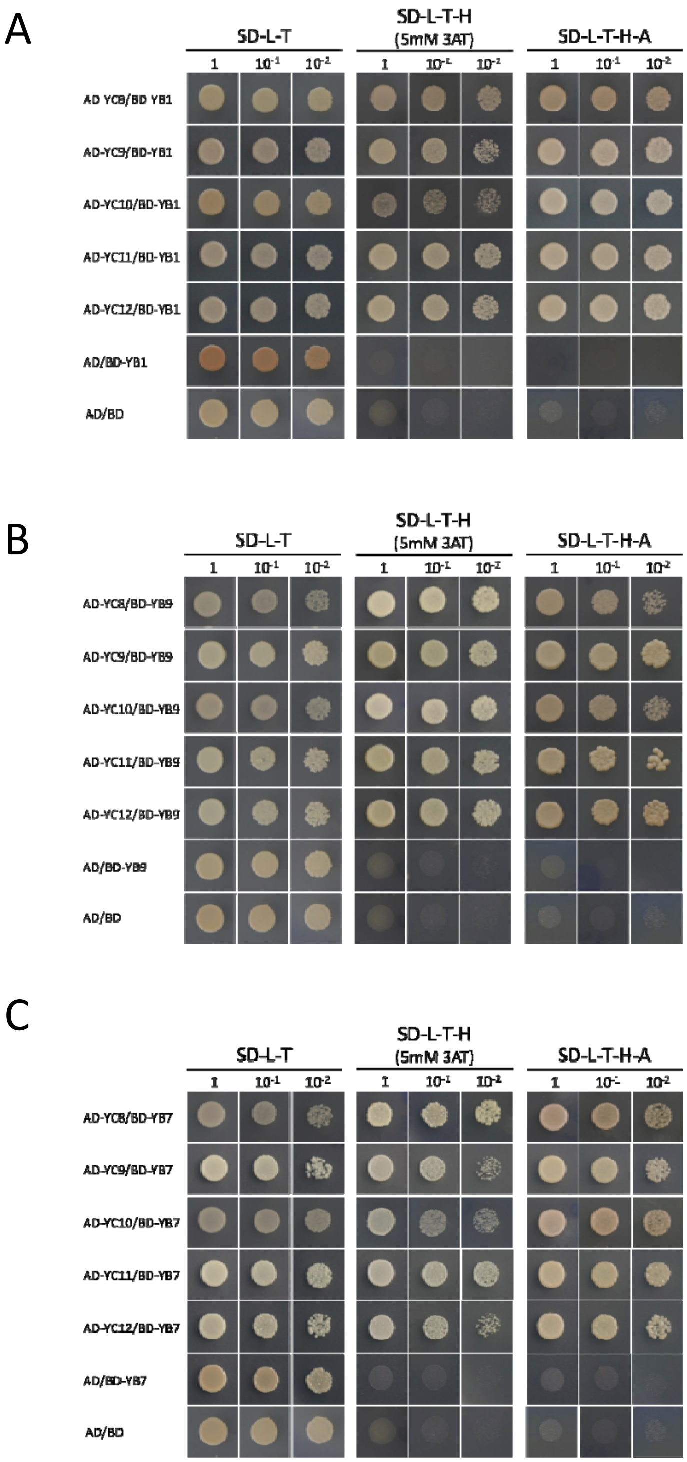

Supplementary Figure 11. OsNF-YB1, OsNF-YB7 and OsNF-YB9 interact with all the endosperm-preferential OsNF-YCs.

(A) Yeast-two-hybrid assay shows OsNF-YB1 can interacts with any of the endosperm-preferential OsNF-YCs. (B) Yeast-two-hybrid assay shows OsNF-YB9 interacts with any of the endosperm-preferential OsNF-YCs. (C) Yeast-two-hybrid assay shows OsNF-YB7 interacts with any of the endosperm-preferential OsNF-YCs. The OsNF-YB1, OsNF-YB7 and OsNF-YB9 were fused with the DNA binding domain of GAL4 (BD), while the endosperm-preferential OsNF-YCs were fused with the activation domain of GAL4 (AD). The yeast cells co-expressed the indicated proteins were plated on the nonselective (SD-L-T) and selective plates (SD-L-T-H with 5mM 3AT and SD-L-T-H-A).

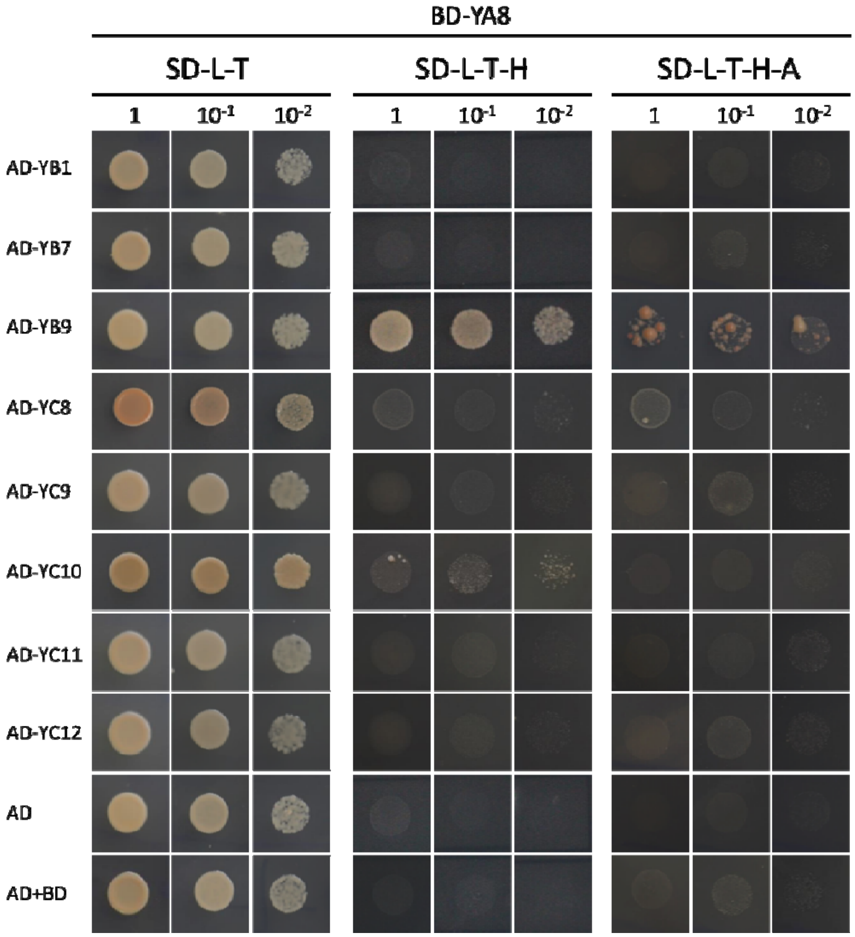

Supplementary Figure 12. OsNF-YA8 interacts with OsNF-YB9, but not with other endosperm-preferential OsNF-Ys.

The OsNF-YA8 was C-terminally fused with the DNA binding domain (BD) of GAL4, while the other endosperm-preferential OsNF-Ys were C-terminally fused with the activation domain (AD) of GAL4. The serially diluted yeast cells expressing the indicated fusions were plated on either the nonselective (SD-L-T) or selective medium (SD-L-T-H and SD-L-T-H-A). Only the transformants co-expressed BD-YA8 and AD-YB9 survived on the selective medium.

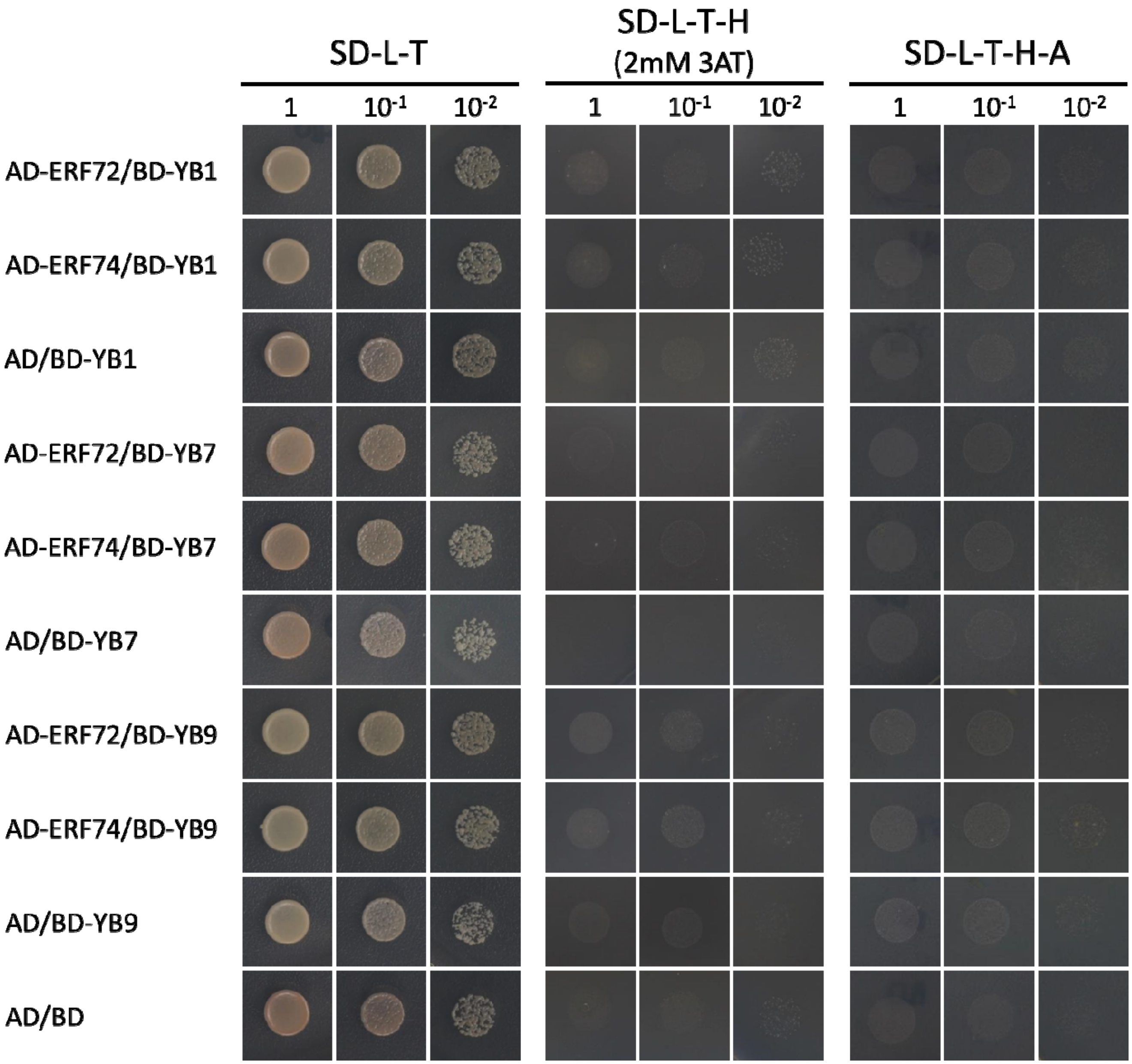

Supplementary Figure 13. OsNF-YB1, OsNF-YB7 and OsYB9 do not show interaction with OsERF72 or OsERF74.

The OsNF-YB1, OsNF-YB7 and OsNF-YB9 were fused with the DNA binding domain of GAL4 (BD), while the OsERF72 and OsERF74 were fused with the activation domain of GAL4 (AD). The yeast cells co-expressed the indicated proteins were plated on the nonselective (SD-L-T) and selective plates (SD-L-T-H with 2mM 3AT and SD-L-T-H-A).

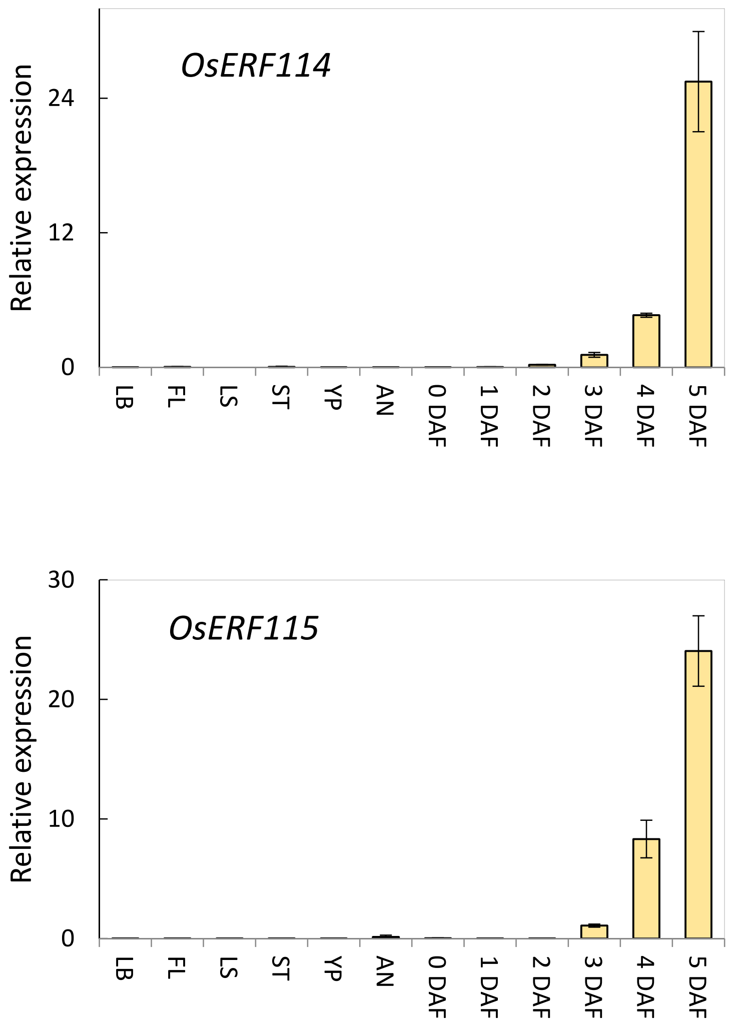

Supplementary Figure 14. *OsERF114* and *OsERF115* are activated after fertilization.
Relative expression of *OsERF114* and *OsERF115* in different tissues were analyzed by real-time PCR assay. LB, FL, LS, YP and AN indicate leaf blade, flag leaf, leaf sheath, young panicle and anther, respectively; 0 to 5 DAF indicate caryopsis of different ages (from 1 days after fertilization to 5 days after fertilization). Three biological replicates were used for analysis. Error bars indicate standard deviations.

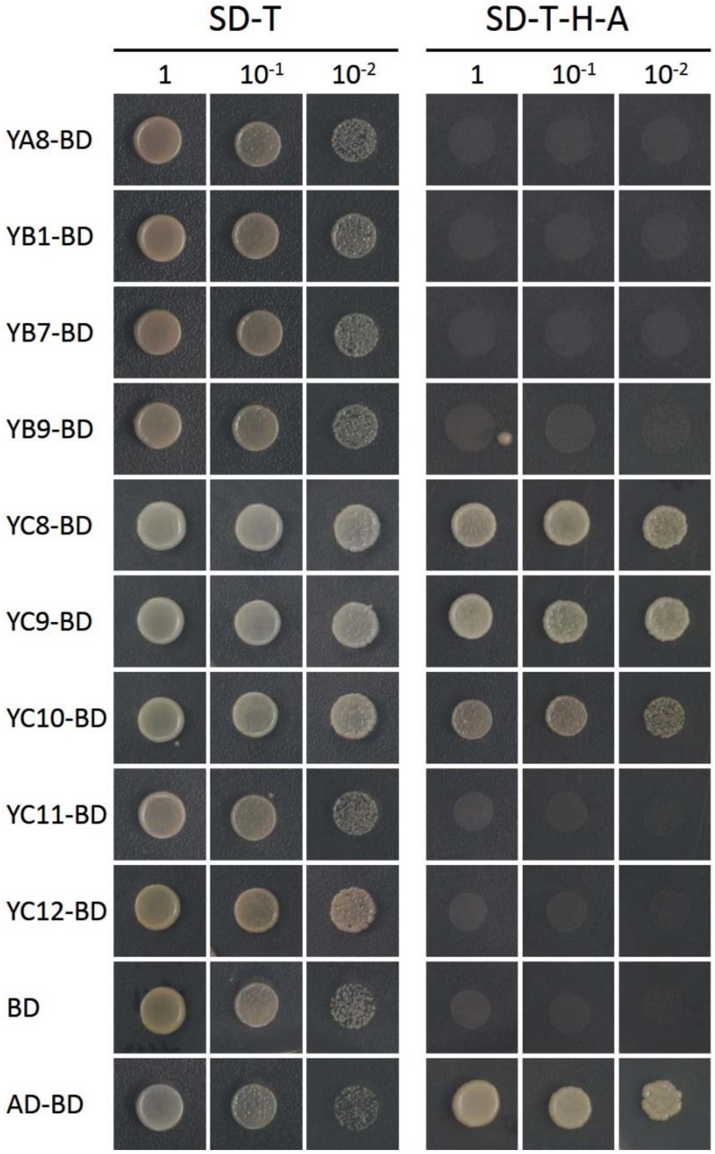

Supplementary Figure 15. OsNF-YC8, OsNF-YC9 and OsNF-YC10 exhibit transcriptional activation activities. The OsNF-YA8, OsNF-YB1,7,9 and OsNF-YC8,9,10,11,12 were cloned into pGBK-T7 (BD) to fuse with the GAL4 binding domain (BD). Empty pGBK-T7 was used as the negative control. The construct that fuse the activation domain of GAL4 (AD) to the binding domain of GAL4 (AD-BD) was used as the positive control. All the constructs grew well on the non-selective medium (SD-T), while only the YC8-BD, YC9-BD, YC10-BD and AD-BD fusions survived on the selective medium (SD-T-H-A), indicating OsNF-YC8,9,10, but not the other NF-Ys we tested, have transcriptional activation activities.

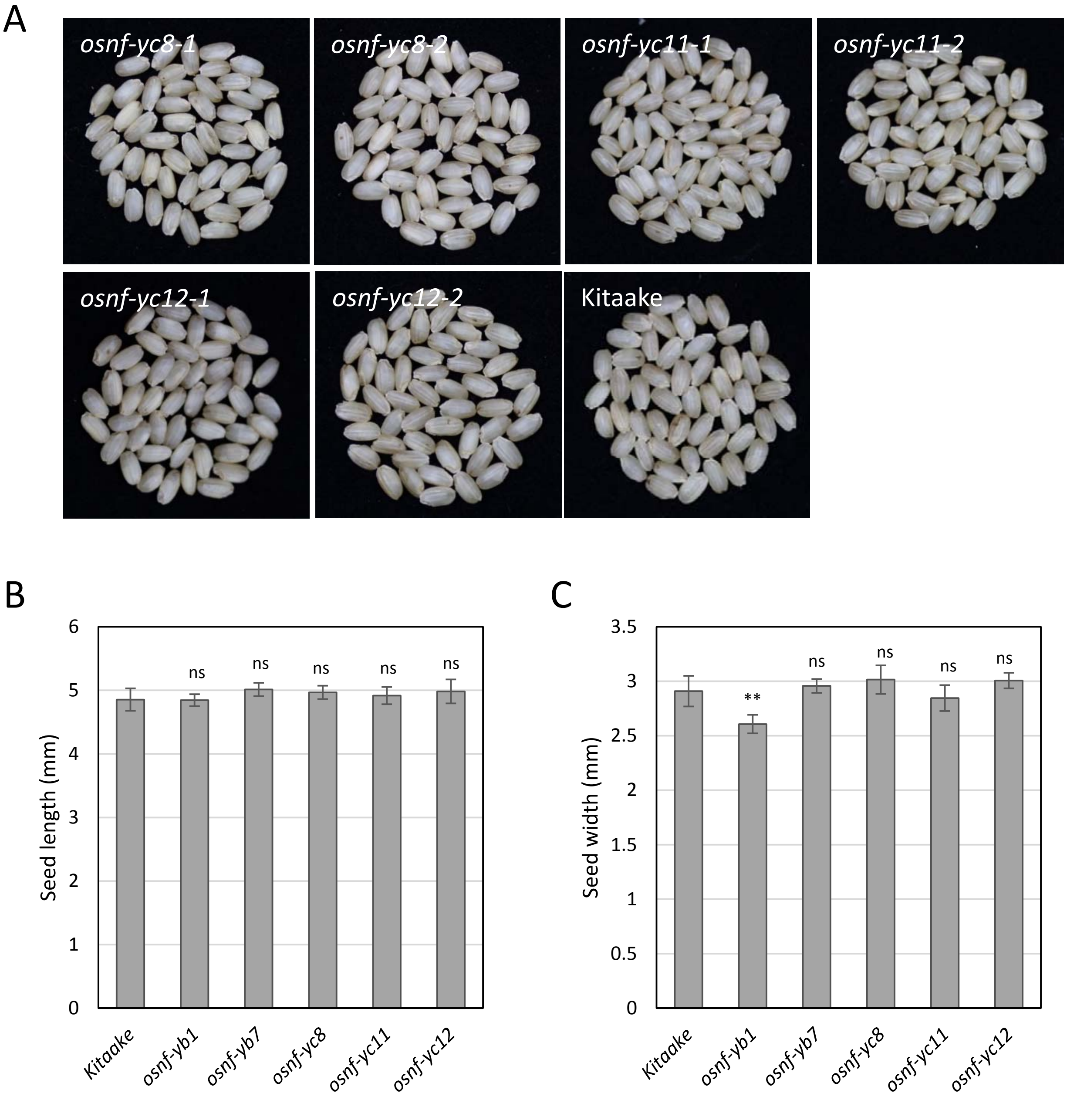

Supplementary Figure 16. Seed morphologies of the seed-preferential *OsNF-Ys* mutants generated by CRISPR/Cas9 approach.

(A) Seed morphology of the homozygous *osnf-yc8*, *osnf-yc11*, *osnf-yc12* T1 mutants and the wild type (WT). Two different mutant alleles for each gene were investigated. **(B-C)** Seed length **(B)** and seed width **(C)** of the *osnf-yb1-1*, *osnf-yb7-1*, *osnf-yc8-1*, *osnf-yc11-1*, *osnf-yc12-1* and the wild type (Kitaake). More than 30 well-filled T0 seeds from the main stem were chose for the measurements; error bars indicate standard deviations. **, *p*<0.01; ns, no significance; t-test was used for the statistical analysis.
